# *Arabidopsis* phospholipid modifications mediate cellulase‐induced resistance to a fungal peptide antibiotic by imposing cell polarity

**DOI:** 10.1111/nph.70721

**Published:** 2025-11-08

**Authors:** Saritha Panthapulakkal Narayanan, Bradley R. Dotson, Lise Noack, Sanjana Holla, Shichao Ren, Peter Dörmann, Susanne Widell, Staffan Persson, Ida Lager, Allan G. Rasmusson

**Affiliations:** ^1^ Department of Biology Lund University Biology Building, Sölvegatan 35B SE‐223 62 Lund Sweden; ^2^ Department of Plant Protection Biology Swedish University of Agricultural Sciences SE‐234 22 Lomma Sweden; ^3^ Copenhagen Plant Science Center (CPSC), Department of Plant & Environmental Sciences University of Copenhagen Thorvaldsensvej 40 1871 Frederiksberg C Denmark; ^4^ Institute of Molecular Physiology and Biotechnology of Plants University of Bonn Karlrobert‐Kreiten‐Strasse 13 53115 Bonn Germany; ^5^ Joint International Research Laboratory of Metabolic & Developmental Sciences, State Key Laboratory of Hybrid Rice, School of Life Sciences and Biotechnology Shanghai Jiao Tong University Shanghai 200240 China; ^6^ Department of Plant Breeding Swedish University of Agricultural Sciences SE‐234 22 Lomma Sweden

**Keywords:** alamethicin, *Arabidopsis*, cellulase, phosphatidic acid, phosphatidylserine, PHOSPHATIDYLSERINE DECARBOXYLASE3, PHOSPHOLIPASE Dζ2, *Trichoderma*

## Abstract

- Plant‐symbiotic *Trichoderma* fungi attack microorganisms by secreting antibiotic membrane‐permeabilising peptaibols such as alamethicin. These peptaibols also permeabilise plant root epidermis plasma membranes (PMs), but mild pretreatment with *Trichoderma* cellulase activates a unique cellulase‐induced resistance to alamethicin (CIRA), via an unknown mechanism.
- We identify two *Arabidopsis* genes that are essential for the CIRA process: *CIRA12* encodes a phosphatidylserine (PS) decarboxylase and *CIRA13*, a phospholipase Dζ, implying that specific changes in anionic membrane lipids mediate alamethicin resistance.
- Fluorescent sensors revealed that cellulase induced a laterally asymmetric decrease in PS and surface charge to outer periclinal root epidermal PMs. Consistently, the CIRA response was reversed by addition of lysoPS. CIRA13 is essential for vesicle trafficking, which in turn is crucial for CIRA induction.
- Overall, cellulase induces a cellular polarity with respect to phospholipids, not previously observed in plants, that is leading to increased lipid packing and preventing peptaibol permeabilization of the outer periclinal membrane.

## Introduction

Yield loss due to phytopathogens is a foremost challenge to agriculture and food security. Therefore, biological agents for crop improvement are being developed (Palmieri *et al*., 2022). Multiple species and strains in the ascomycete fungal genus *Trichoderma* are heavily used as direct biostimulators and as biological control agents against various plant pathogens in eco‐sustainable agriculture (Harman *et al*., 2004; Druzhinina *et al*., 2011; Woo *et al*., 2014, 2023). However, the mechanisms of plant–*Trichoderma* interaction, and especially plant genetic factors that govern successful symbiosis are all but clear. *Trichoderma‐*induced growth enhancement in tomato (Tucci *et al*., 2011), lentil (Prashar & Vandenberg, 2017), sugar beet (Schmidt *et al*., 2020) and maize (Harman, 2006) depends on the plant genotype. So, although *Trichoderma* is generally a beneficial fungus, not all plant–*Trichoderma* interactions have positive outcomes, and the lack of mechanistic understanding prevents prediction of genotype‐specific *Trichoderma* effects.

*Trichoderma* produces many potential signalling molecules and effectors such as plant‐recognised hormones and microbe‐associated molecular patterns, which activate various plant defence pathways such as systemic acquired resistance, induced systemic resistance and different growth regulation pathways (Hermosa *et al*., 2012; Alfiky & Weisskopf, 2021). However, symbiotic fungi also produce cell wall degrading enzymes, antimicrobial metabolites, volatile organic compounds and reactive oxygen species that attack other microbes as part of antibiosis and mycoparasitism (Mendoza‐Mendoza *et al*., 2018). A major class of antimicrobial peptides (AMPs) secreted by *Trichoderma* are peptaibols, which cause membrane permeabilization and cell lysis (Duclohier, 2010; Druzhinina *et al*., 2011). Peptaibols are linear amphipathic peptides consisting of 5–20 mainly nonproteinogenic residues. The most studied peptaibol is the 20‐residue alamethicin from *Trichoderma viride*. It inserts into energised membranes when approaching from the net positively charged side, forming pores that lyse cells (Duclohier & Wroblewski, 2001; Leitgeb *et al*., 2007; Duclohier, 2010). When alamethicin is added to sterile‐cultured tobacco cells, alamethicin permeabilises the plasma membrane (PM), and, in turn, mitochondria and chloroplasts, leading to the release of metabolites and coenzymes (Matic *et al*., 2005; Aidemark *et al*., 2009), and subsequently apoptosis‐like cell death (Rippa *et al*., 2010). However, a mild pretreatment of the cells with cellulase from *Trichoderma* induces plant cell resistance to this peptaibol (Aidemark *et al*., 2010); a process named cellulase‐induced resistance to alamethicin (CIRA) (Dotson *et al*., 2018). This process is unique as an induced defence against a peptaibol by a eukaryotic cell. Eukaryotes produce AMPs, such as thionin, defensin and cathelicidin, to act against invading microbial pathogens (Zasloff, 2002; Cederlund *et al*., 2011; Tam *et al*., 2015). Different classes of membrane‐active AMPs (maAMPs) have attracted interest for use in medical therapeutics and for drug delivery (Mahlapuu *et al*., 2016; Buda De Cesare *et al*., 2020; Datta & Roy, 2021; Hadjicharalambous *et al*., 2022), for which cellular specificity of permeabilization is crucial, yet induced changes in eukaryotic resistance against AMPs have not been reported. By contrast, Gram‐positive bacteria such as *Staphylococcus* spp., *Streptococcus* spp. and *Enterococcus* spp. can mount induced and/or constitutive defence against maAMPs released by human cells (Frick *et al*., 2003; Samuelsen *et al*., 2005; Majchrzykiewicz *et al*., 2010; Saar‐Dover *et al*., 2012; Simanski *et al*., 2013; Gebhard *et al*., 2014; Hirt *et al*., 2018; Khan *et al*., 2019). Although maAMP resistance by secretion of specific proteases and virulence factors can occur, modifications of the bacterial membranes are the most common resistance mechanisms (Mahlapuu *et al*., 2016; Assoni *et al*., 2020; Datta & Roy, 2021). During the CIRA induction in tobacco cells, a modification of the PM composition was observed, including a decreased ratio of sterol to membrane phospholipids and a lower level of anionic phospholipids (Aidemark *et al*., 2010).

The major anionic phospholipids phosphatidylserine (PS), phosphatidic acid (PA) and phosphoinositides (PIs) represent a minor percentage of lipids in the PM of *Arabidopsis thaliana* (Noack & Jaillais, 2020). PS accumulates in the cytosolic leaflet of PM and is required for multiple processes in plant roots, including cell plate formation (Yamaoka *et al*., 2021) and regulation of Rho GTPase signalling in auxin recognition (Platre *et al*., 2018, 2019). PS is synthesised from phosphatidylethanolamine (PE) in the endoplasmic reticulum (ER) by PS SYNTHASE 1 (PSS1) (Yamaoka *et al*., 2011), and can again be converted to PE by PS decarboxylase (PSD) enzymes (Vance, 1990). The *Arabidopsis* PSD family includes PSD1 localised in mitochondria, and PSD2 and PSD3 that accumulate in endomembranes, likely to the tonoplast and the ER, respectively (Nerlich *et al*., 2007). *PSD1* and *PSD2* are highly expressed in flowers, whereas *PSD3* is also expressed in roots, stems and leaves (Nerlich *et al*., 2007). Even though the *psd1*, *psd2* and *psd3* mutants have reduced *PSD* activity, they have no reported phenotypic deviations from wild‐type (WT) during normal cultivation (Nerlich *et al*., 2007).

PA can be formed primarily through the hydrolysis of phosphatidylcholine (PC) by phospholipase D (PLD) enzymes (Hornberger *et al*., 2006). Additionally, PA can be synthesised by the enzymes, diacylglycerol (DAG) kinase (Ciprés *et al*., 2003) and lysophosphatidic acid acyl *trans*ferase (Bradley & Duncan, 2018). Among these enzymes, DAG kinase and PLDs are the major enzymes producing signalling PA (Yao *et al*., 2025). Generally, PLDs are classified into C_2_‐PLDs and PH/PX‐PLDs based on protein domain structures (Wang, 2005). C_2_‐PLDs contain Ca^2+^ and phospholipid‐binding domains and convert PC, PE and PG to PA, whereas PH/PX‐PLDs comprise distinct structural folds of pleckstrin (PH) and phox homology (PX) and are selective for PC (Wang, 2005; González‐Mendoza *et al*., 2021). Of the 12 *Arabidopsis* PLDs, 10 belong to C_2_‐PLDs (PLDα‐ε) and two are grouped into PH/PX‐PLDs (PLDζ1 and PLDζ2) (Wang, 2005). Both C_2_‐PLDs and PH/PX‐PLDs are involved in biotic and abiotic stress tolerance (Li *et al*., 2009).

Phenotypes of *Arabidopsis* plants deficient in or overexpressing particular PLDs have indicated that each PLD is involved in a unique plant function or response (Wang, 2005; Li *et al*., 2009; Hong *et al*., 2016). The different C_2_‐PLDs have also been shown to vary considerably in subcellular localization, including homologues residing in the cytosol, nucleus or mitochondria, or being bound to PM, intracellular membranes, actin or microtubule (Fan *et al*., 1999; Takáč *et al*., 2019; Wang, 2001; Hong *et al*., 2008, 2009). Among the PH/PX‐PLDs, PLDζ1 has been localised to the *trans*‐Golgi network (TGN) and PLDζ2 to the tonoplast, TGN and multivesicular bodies (MVBs) (Yamaryo *et al*., 2008; Shimamura *et al*., 2022). In plants, the TGN serves as an early endosome directing endocytosed transmembrane proteins to vacuoles and PM (Uemura, 2016). MVBs serve as late endosomes directing endocytosed cargos from TGN to the vacuole (Cui *et al*., 2016). PLDζ2 activates vesicle trafficking (Li & Xue, 2007), most likely regulating the distribution of vesicles to the PM and/or the tonoplast via MVB and the TGN (Shimamura *et al*., 2022).

Similar to tobacco cells, alamethicin permeabilization and the CIRA response occurs in *Arabidopsis* root tip epidermis cells, especially in the basal meristematic and extension zone (Dotson *et al*., 2018). However, the mechanism behind alamethicin resistance is unexplored. In this study, we identified a cellulase‐induced lateral asymmetry, that is an unequal distribution of phospholipids along the PM, resulting in a cell polarity of the root epidermis PM, in which changes in surface charge specific to the outer periclinal PM domain results in CIRA. This study also exposes the vital role of vesicle trafficking for CIRA efficacy.

## Materials and Methods

### Plant materials and growth conditions

*Arabidopsis thaliana* (L.) Heynhold WT and the mutants – *cira12‐1* (SALK_055283C), *cira12‐3* (SALK_015024), *cira13‐1* (SALK_012466C), *cira13‐2* (SALK_119084C) (Li & Xue, 2007), *cira13‐3* (SALK_094369) (Yamaryo *et al*., 2008; Shimamura *et al*., 2022), *pldz1* (SALK_083090C) (Yao *et al*., 2013), *psd2* (SALK_ 038354), *pss1* (GABI_166G10) (Platre *et al*., 2018), mCIT‐C2^LACT^, mCIT‐1XPASS and mCIT‐KA1^MARK1^ used in this study are in the Columbia‐0 (Col‐0) background. All mutants were confirmed by polymerase chain reaction (PCR) and reverse transcription PCR (RT‐PCR) using primers as denoted in Supporting Information Table S1. For RT‐PCR, RNA was isolated using the RNeasy Plant Mini Kit (Qiagen), and the cDNA was synthesised using Maxima First Strand cDNA Synthesis Kit (ThermoScientific, Waltham, MA, USA).

*Arabidopsis* seeds were surface‐sterilised with 70% (v/v) ethanol for 1 min followed by 50% (v/v) household bleach containing 0.1% Tween 20 for 1 min, washed with distilled water five times and incubated at 4°C for 2 d. For assays using primary roots, 5–10 sterilised seeds were transferred to each well of 96‐well polypropylene (PP) plates with lids (Greiner Bio‐One, Kremsmünster, Austria), and 100 μl of water or ½ Murashige & Skoog (MS) liquid medium (Murashige & Skoog, 1962) was added. The plates were transferred to a growth room (16‐h light, 22–24°C). These seedlings were used for CIRA assays after 6 d of growth.

For assays using mainly lateral roots, the sterilised seeds were plated on ½ MS agar plates and grown vertically. After 14 d, the seedlings were transferred to a 96‐well PP plate (GreinerBio‐One), having two seedlings per well in 100 μl of water. These seedlings were used for CIRA assays. It is essential to grow seedlings in PP plates, because according to our observations, several brands of polystyrene plates will render the root tips ultrasensitive to cellulase, leading to visually observable damages, yet variably between batches from the same brands.

### CIRA assay

After growth, the medium surrounding the seedlings was replaced with the same volume of water ±1% (w/v) cellulase (pH 5, ONOZUKA RS, Duchefa, Haarlem, the Netherlands) and incubated on a shaker for 2 h. After incubation, the seedlings were washed three times with 200 μl water. Then, 10 or 20 μg ml^−1^ (see the data presentation legends) alamethicin (A‐4665 ‘from *T. viride*’, Sigma‐Aldrich (St. Louis, MO, USA)) was added to the seedlings and incubated for 10 min. Although this strain of *T. viride* has been renamed as *T. arundinaceum* (Degenkolb *et al*., 2008), it is still identified as *T. viride* by the producer. Controls contained an identical concentration of ethanol. For fluorescence analyses, the seedlings were then stained for 1 min by supplementation with 1.5 μM PrI. Solution from the wells was transferred to a new plate for ionic conductivity measurement using a CDM230 Conductivity Meter (Radiometer, Brønshøj, Denmark). Electrode fouling due to alamethicin was corrected for by dividing the conductivity per seedling by the relative conductivity at the same alamethicin concentration without seedlings. The concentration of cellulase used in this study for CIRA assays led to a mild degradation of the cell wall that was hardly visible by microscopy, as previously documented (Dotson *et al*., 2018).

Fluorescence microscopy was performed on the seedlings using a G‐2A filter (excitation at 510–560 nm, emission above 590 nm) coupled to a Nikon Optiphot‐2 microscope (Nikon Corporation, Tokyo, Japan). A bright‐field transmission microscopy image was taken as a reference. Images were captured using an Olympus DP‐70 digital camera (Olympus Optical, Tokyo, Japan). In each experiment, the exposure times were selected from control roots treated with alamethicin, and the same setting was then used for capturing all images within the experiment.

### Lysophospholipid treatments

Seedlings grown as mentioned previously were washed with water and treated with cellulase for 1 h with gentle agitation. Thereafter, the cellulase solution was supplemented with 54 μM lysophospholipid – lysoPC (Avanti Polar Lipids 845 875, AL, USA), lysoPE (Avanti Polar Lipids 846 725), lysoPA (Avanti Polar Lipids 857 130) or lysoPS (Avanti Polar Lipids 858 143). Seedlings were further incubated with gentle agitation for 1 h. The seedlings were washed three times with 200 μl water and then 100 μl of 10 μg ml^−1^ alamethicin was added to the seedlings and incubated for 10 min. The solution from the wells was then removed and analysed for changes in conductivity.

### Inhibitor treatments

Seedlings grown as mentioned previously were washed with water and treated overnight with 300 nM PLDζ inhibitors VU1 (VU0359595 Avanti Polar Lipids), and/or VU2 (VU0285655‐1 Avanti Polar Lipids) in ½ MS (procedure modified from Yao *et al*., 2013) (Yao *et al*., 2013). For the exocytosis inhibitor brefeldin A (BFA) (Sigma‐Aldrich B6542, Merck Life Science AB, Solna, Sweden) treatment, the seedlings were washed with water and treated with 36 μM BFA in ½ MS for 30 min with gentle agitation. For endocytosis inhibitor wortmannin (WM) (Sigma‐Aldrich W1628) treatment, the seedlings were washed and treated with 33 μM WM in ½ MS for 1 h with gentle agitation. For PA (DAG kinase inhibitor‐R59949), PI4P inhibitor (PAO), tyrphostin A (T) and endosidin 2 (E) treatments, the seedlings were washed with water and treated with 12.5 μM R59949, 30 μM PAO, 30 μM T or 40 μM E, in ½ MS for 1 h (R59949 and PAO), 30 min (T) or 2 h (E) with gentle agitation (Platre *et al*., 2018). After respective inhibitor treatments, the seedlings were washed with water and incubated for 2 h with cellulase on a shaker with gentle agitation. The seedlings were then washed thrice with water, and 10 μg ml^−1^ alamethicin was added to the seedlings and incubated for 10 min. Solution from the wells was analysed by ionic conductivity measurement.

### Confocal microscopy

Confocal microscopy imaging experiments were performed on 6‐d‐old seedlings expressing the PS, PA or anionic phospholipid reporters mCIT‐C2^LACT^, mCIT‐1XPASS and mCIT‐KA1^MARK1^ (Platre *et al*., 2018), respectively, which were then treated with and without cellulase. The mCITRINE fluorescence was detected using a spinning disc confocal microscope set‐up containing an inverted Zeiss microscope (AxioObserver Z1, Carl Zeiss Group, Oberkochen, Germany) equipped with a spinning disc module (CSU‐W1‐T3, Yokogawa, Amersfoort, the Netherlands) and a ProEM+ 1024B camera (Princeton Instrument, Krailing, Germany) using a 63× objective (oil immersion). The mCITRINE was excited with a 515‐nm laser and detected at 520 nm and above. All fluorescent images presented were acquired using the same exposure settings and processed equally.

### Statistical analysis

Statistical analyses were carried out using Student's *t*‐test (denoted by asterisks) or one‐way analysis of variance (ANOVA) followed by Tukey's *post hoc* analysis (denoted by letters).

## Results

### Cellulase‐induced resistance to alamethicin permeabilization is lost in *cira12* and *cira13* mutants

**Fig. 1:**
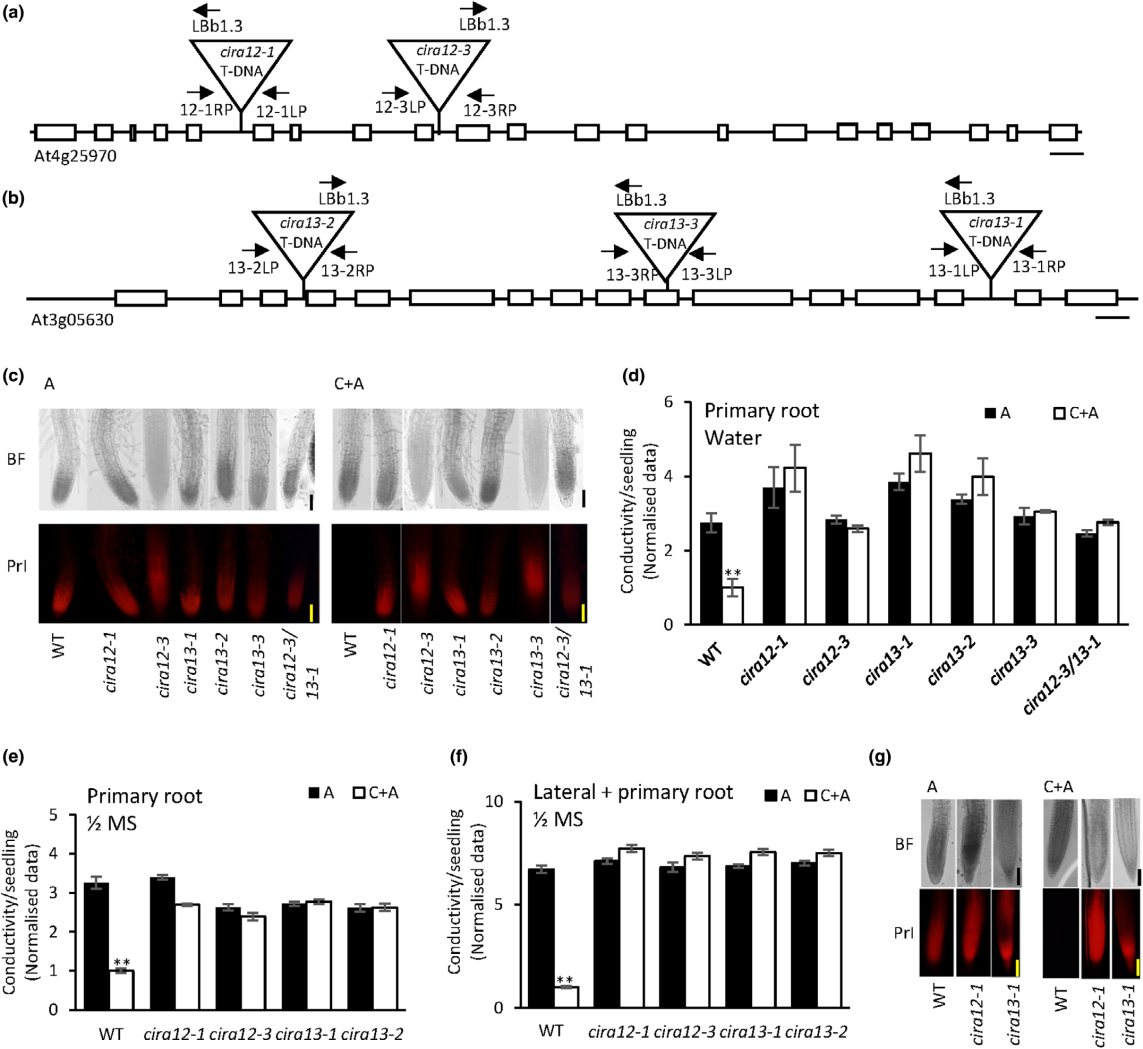
Identification of mutants preventing cellulase‐induced resistance to alamethicin (CIRA) in primary and lateral roots. Schematic map of the T‐DNA insertions in (a) *CIRA12* (At4g25970) and (b) *CIRA13* (At3g05630) genomic sequences (Bar, 150 bp). White boxes represent exons. LBb1.3, SALK T‐DNA border primer; LP, left primer; RP, right primer. (c, d) Cellulase‐induced resistance to subsequent alamethicin permeabilization in wild‐type (WT) *Arabidopsis* seedlings is lost in *cira12* and *cira13* mutants. Seedlings of WT and *cira* mutants were incubated in cellulase (C) followed by alamethicin (A) (20 μg ml^−1^) and propidium iodide (PrI). (c) Fluorescent microscopy images revealing loss of CIRA in *cira* mutants. (d) Detection of CIRA in WT and *cira* mutants by measuring ion leakage. Detection of CIRA in primary (e) and lateral (f) root tips in WT and *cira* mutants was analysed as ion leakage. Seedlings were grown in ½ Murashige & Skoog (MS) medium for 6 d (e) for primary root assays, and 14 d (f) for lateral root assays, incubated in cellulase followed by alamethicin (10 μg ml^−1^) and PrI. Ten to 12 lateral roots were observed per seedling. Conductance data were normalised by dividing by control (WT (C + A)), the average of which was 1.05 ± 0.04, 0.77 ± 0.02, and 8.13 ± 0.08 μS cm^−1^ per seedling (mean ± SE) in (d), (e) and (f), respectively. (g) Fluorescent microscopy images of lateral roots of WT and each *cira* mutant showing alamethicin permeabilization. Microscopy images show one representative replicate out of three. Bars, 100 μm. BF, bright field. Asterisks denote significant difference (**, *P* < 0.01) after cellulase pre‐treatment as compared to control. Error bars show SE of the mean (*n* = 3). Statistical analyses were carried out using Student’s *t*‐test.

Application of alamethicin to 6‐d‐old water‐grown *Arabidopsis* seedlings led to cell permeabilisation in the epidermis of the root extension zone as observed by cell entry of the DNA‐intercalating fluorescent probe propidium iodide (PrI). However, this was prevented by a 2‐h pretreatment with 1% *Trichoderma* cellulase inducing CIRA (Fig. 1c,d) (Dotson *et al*., 2018). In an ongoing screen for T‐DNA mutants deficient in CIRA, we obtained two *cira* mutants that affected genes associated with lipid metabolism, namely CIRA12 and CIRA13. When WT and the CIRA mutants were treated with cellulase followed by alamethicin to detect CIRA, only the WT seedlings displayed the absence of PrI uptake and fluorescence, typical for the CIRA response (Fig. 1c). The interruption and lack of expression of PSD3 (CIRA12) and PLDζ2 (CIRA13) in *cira* mutants – *cira12‐1*, *cira12‐3*, *cira13‐1*, *cira13‐2* and *cira13‐3* were shown by PCR genotyping and RT‐PCR (Fig. S1). We developed a CIRA assay that measures ion leakage from *Arabidopsis* seedlings caused by the alamethicin permeabilization in microtitre plates at semihigh throughput. In this way, we determined the concentration dependence of alamethicin on ion leakage with and without cellulase pretreatment. We found a steady increase in conductivity at 10 μg ml^−1^ alamethicin (Fig. S2) and used this concentration in our conductivity‐based CIRA analysis assays. When seedlings were pretreated with cellulase, the alamethicin‐dependent ion release was significantly lower in WT seedlings than in the noncellulase‐treated control (Fig. 1d). By contrast, the *cira* mutants did not show alterations in conductivity ± cellulase pretreatment, confirming their loss of the CIRA response (Fig. 1d). A double‐mutant *cira12‐3/cira13‐1* gave similar results as corresponding single mutants for both fluorescence‐based and conductivity‐based CIRA detection (Fig. 1c,d). Since the results from fluorescent microscopy and conductivity measurement were fully consistent, we mainly used the conductivity‐based assay to detect CIRA.

The CIRA12 and CIRA13 proteins are both involved in conversions between anionic (PS, PA) and net uncharged (zwitterionic) phospholipids. CIRA12 (At4g25970) is PSD3 that decarboxylates PS to PE in the ER/Golgi. CIRA13 (At3g05630) is PLDζ2 that produces PA from PC in Golgi and tonoplast (Dubots *et al*., 2012). To see whether CIRA12 and 13 homologues may also be associated with CIRA, we performed CIRA assays on *PSD2* (At5g57190) and *PLDζ1* (At3g16785), respectively. However, *psd2* and *pldζ1* behaved similar to WT, suggesting that these genes are not involved in CIRA (Fig. S3e), and that CIRA12/PSD3 and CIRA13/PLDζ2 therefore have distinct functions in the CIRA response. We speculated that CIRA12 may act by decreasing the abundance of PS in the PM and thus its surface charge. To test whether general changes in PS affect CIRA, we also analysed homozygous *pss1* (At1g15110) mutant plants (Fig. S3f), which are deficient in PS (Platre *et al*., 2018). The *pss1* mutant displayed a CIRA response as WT, indicating that changes in other charged phospholipids such as PA or PIs may cause CIRA in the *pss1* mutant. However, external application of DAG kinase and PI4P formation inhibitors did not affect alamethicin‐induced ion release in WT seedlings, and a slight inhibition of ion release was only observed in the *cira* mutants when treated with both inhibitors together (Fig. S4). This indicates that changes in DAG kinase and PI4P formation have little effect on CIRA in WT and *cira* mutants. Furthermore, PS is known to bind the Rho of Plants 6 (ROP6) protein, forming nanodomains in the PM, which are necessary for auxin signalling (Platre *et al*., 2019). Therefore, we also tested mutants for five ROP genes, including ROP6. None of these affected the CIRA process (Fig. S3g).

Initial experiments were carried out with seedlings grown in milli Q water. However, under phosphate (P_i_) starvation, there is a decrease in the level of membrane phospholipids (PC, PE, PI and PA) in *Arabidopsis* roots (Li *et al*., 2006b). Therefore, CIRA mutant phenotypes were also analysed in seedlings grown for 6 d in ½ MS, which contains 0.5 mM P_i_. In this P_i_‐repleted growth condition, WT seedlings displayed CIRA, whereas mutant genotypes showed a loss of CIRA (Fig. 1e). To test CIRA and mutation effects also in lateral roots, we monitored CIRA responses in 2‐wk‐old seedlings, each with 10–12 lateral root tips (Fig. 1f). The result showed a lateral root response that was similar to that of primary roots for both WT and *cira* mutant seedlings (Fig. 1g). Taken together, the results indicate that the CIRA12‐/CIRA13‐dependent peptaibol resistance process is active in both primary and lateral root tips of WT seedlings, irrespective of low or high phosphate conditions.

### Cellulase treatment decreases surface charge in the outer periclinal PM, inducing electrical cell polarity

**Fig. 2:**
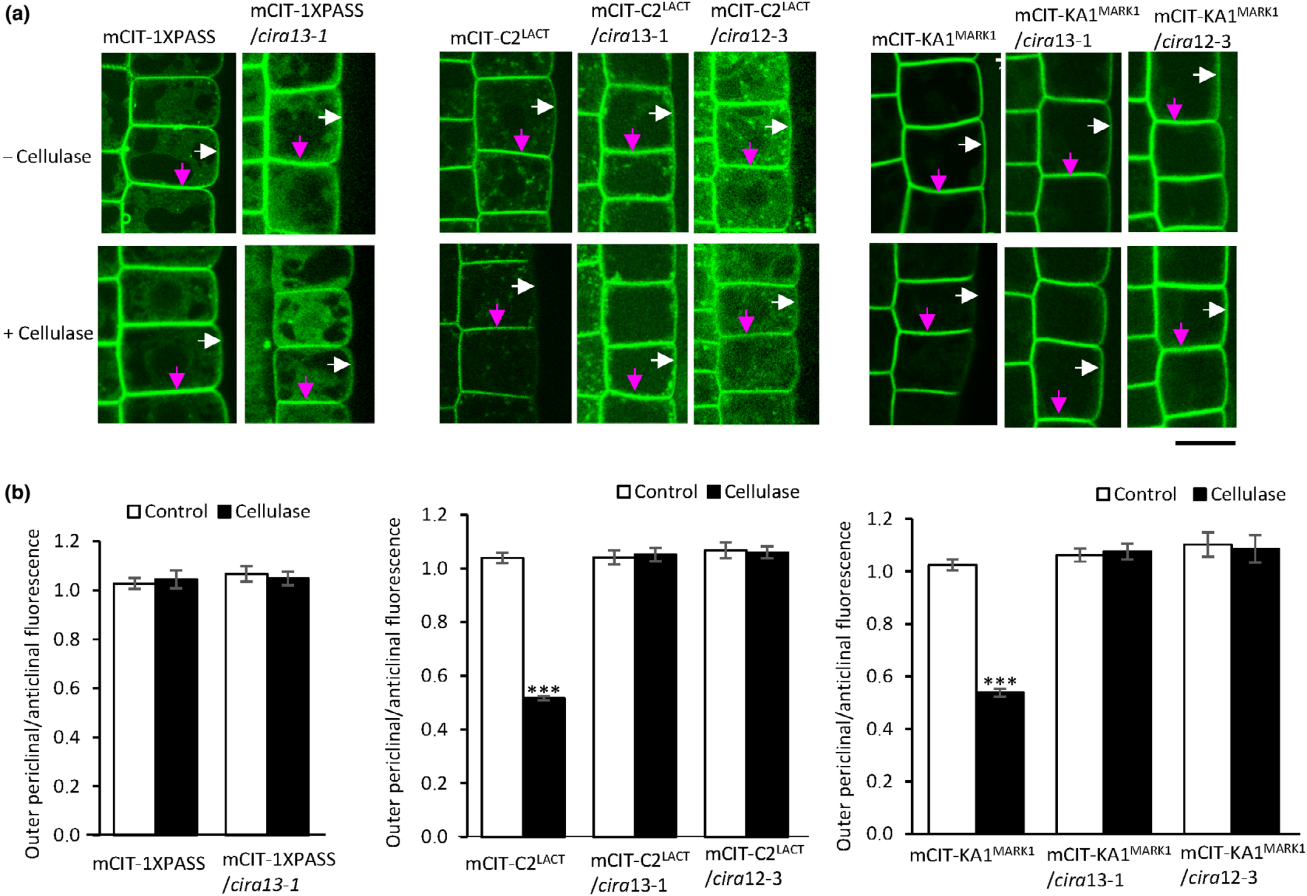
Cellulase‐induced lateral asymmetry is observed in the plasma membrane of the wild‐type but not in *cellulase‐induced resistance to alamethicin* (*cira*) mutants. (a) Root early extension zone epidermis of *Arabidopsis* wild‐type (WT) and mutant seedlings *cira13‐1* expressing the mCIT‐tagged distribution sensors for phosphatidic acid (PA) (mCIT‐1XPASS), phosphatidylserine (PS) (mCIT‐C2^LACT^) and anionic phospholipids (mCIT‐KA1^MARK1^), and *cira12‐3* expressing mCIT‐C2^LACT^, mCIT‐KA1^MARK1^ treated with and without cellulase were analysed by spinning disc confocal microscopy. Magenta arrows highlight dual anticlinal plasma membranes (PMs) separated by a cell wall; white arrows indicate the outer periclinal PM. Bar, 10 μm. (b) Quantified PA, PS and anionic phospholipid fluorescence intensity ratio between outer periclinal and anticlinal PM domains in root epidermal cells with and without cellulase treatment. Asterisks denote significant difference (***, *P* < 0.001) in the ratio of fluorescence intensity between cellulase‐pretreated and control. Error bars show SE of the mean (*n* = 30). Ratios have been corrected for the fact that the anticlinal signal comes from two membranes in close proximity. Statistical analyses were carried out using Student’s *t*‐test.

Because CIRA12 and CIRA13 both modify phospholipids, we next analysed the effect of cellulase on the endogenous anionic lipids PS and PA in epidermal root cell PM. The CIRA response should only demand membrane lipid changes in a few cells in the root, and lipid profiles for *psd3 (cira12)* and *pldζ2 (cira13)* have been reported to be similar to WT (Li *et al*., 2006b; Nerlich *et al*., 2007), so we took the approach of using spinning disc fluorescent microscopy on WT and mutant lines expressing the PS‐ and PA‐specific mCITRINE‐tagged lipid distribution sensors mCIT‐C2^LACT^ and mCIT‐1xPASS, respectively (Platre *et al*., 2018). The PA sensor was shown to localise at the PM and in the cytosol and the PS sensor at the PM and intracellular membranes under normal growth conditions (Platre *et al*., 2018). After cellulase treatment, we observed an asymmetric change in the PS sensor fluorescence, that is reduced fluorescence in the outer periclinal PM as compared to the anticlinal PM in WT seedlings (Figs 2, S5a). A twofold decrease in the ratio of PS between outer periclinal and anticlinal PM was observed with the PS sensor in the WT background after cellulase treatment (Fig. 2b). By contrast, no consistent change in the distribution of PA was observed upon cellulase treatment (Figs 2, S5a). Seeing a change in PS, we also analysed the surface potential of the PM using the mCITRINE‐tagged reporter for anionic membrane phospholipids mCIT‐KA1^MARK1^ (Platre *et al*., 2018). Similar to PS distribution, the surface charge ratio between outer periclinal and anticlinal PM was decreased twofold in response to cellulase treatment in WT seedlings (Fig. 2b). By contrast, confocal microscopy on *cira13‐1* expressing mCIT‐1xPASS, mCIT‐C2^LACT^, mCIT‐KA1^MARK1^ and *cira12‐3* expressing mCIT‐C2^LACT^, mCIT‐KA1^MARK1^ did not display a difference in distribution between outer periclinal and anticlinal PM, with or without cellulase treatment (Figs 2, S6a). Seedlings of WT and *cira* mutants expressing mCIT‐1xPASS, mCIT‐C2^LACT^ and mCIT‐KA1^MARK1^ did not show a significant difference in surface charge ratio between cytoplasm and anticlinal PM, indicating no correlation between the treatment and the variation in cytoplasmic signal (Figs S5b, S6b). Hence, these results indicate that cellulase treatment specifically decreases the level of PS, but not PA, in the outer periclinal PM of root epidermal cells of WT *Arabidopsis* leading to a change in surface charge.

### Addition of LysoPS counteracts CIRA

**Fig. 3:**
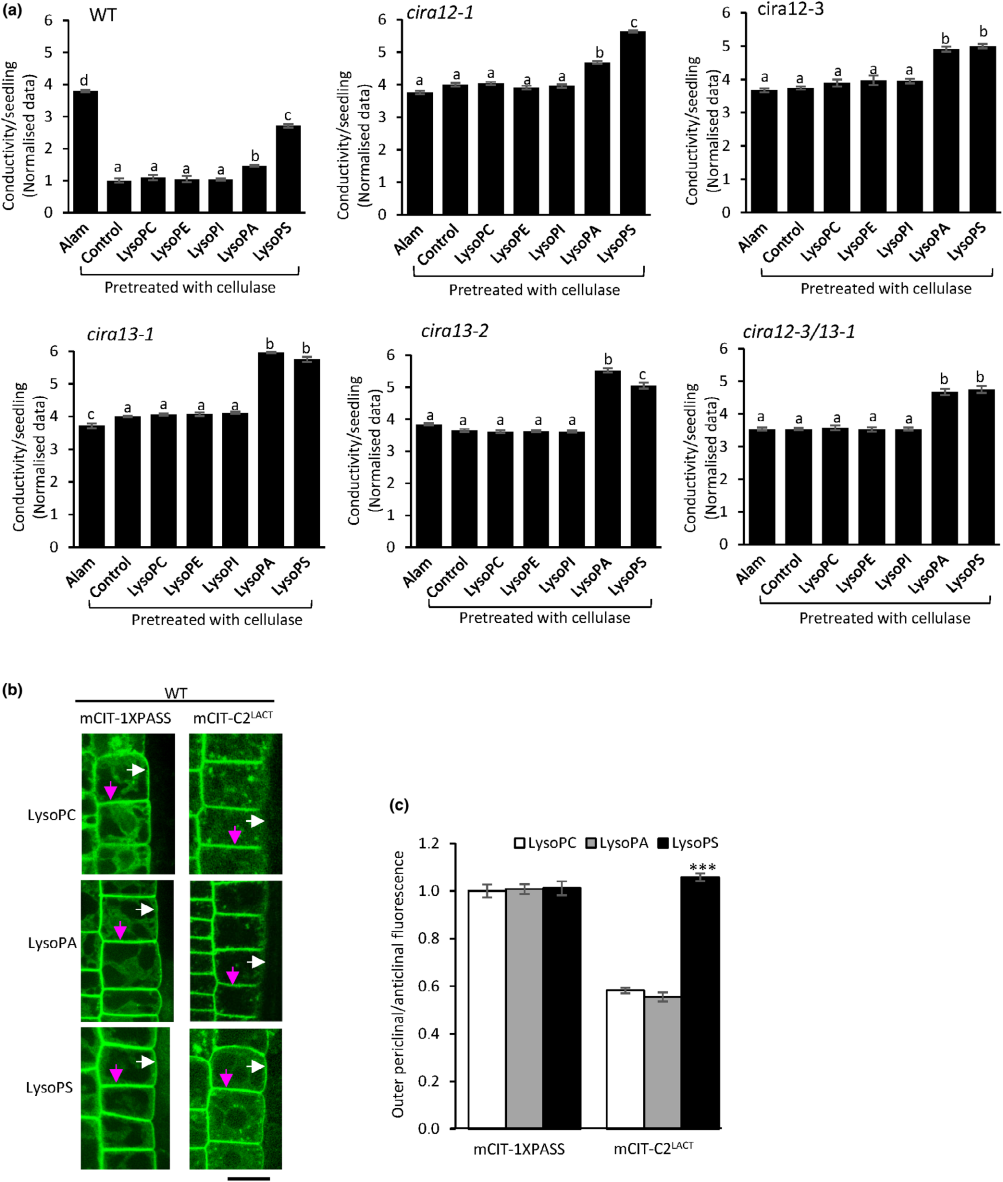
External application of anionic phospholipid in lyso‐form counteracts cellulase‐induced resistance to alamethicin (CIRA) in wild‐type (WT). (a) WT and *cira* mutants were pretreated with cellulase and various lysophospholipids and thereafter tested for alamethicin‐dependent ion release. Loss of CIRA was specifically observed in WT seedlings post lysophosphatidic acid (lysoPA) and even more so post lysophosphatidylserine (lysoPS) treatment. Seedlings pretreated with cellulase plus solvent followed by alamethicin were used as controls. Bar with alamethicin (Alam) treatment alone is included to show the extent of loss of CIRA response by lysophospholipid treatments. Data were normalised by dividing by the mean of the WT control, the average of which was 0.53 ± 0.04 μS cm^−1^ per seedling (mean ± SE). Values marked by the same letter are not statistically different (*P <* 0.05). Statistical analyses were carried out using one‐way analysis of variance (ANOVA) followed by Tukey *post‐hoc* analysis (denoted by letters). (b, c) Cellulase‐induced lateral phosphatidylserine (PS) asymmetry in the plasma membrane (PM) is reversed by lysoPS. (b) Root early extension zone epidermis of WT *Arabidopsis* seedlings expressing distribution probes mCIT‐1XPASS, mCIT‐C2^LACT^ treated with cellulase and lysoPC/lysoPA/lysoPS were analysed by spinning disc confocal microscopy. LysoPC treated seedlings were used as controls. Magenta arrows highlight dual anticlinal PMs separated by a cell wall; white arrows indicate the outer periclinal PM. Bar, 10 μm. (c) Quantified mCIT‐1XPASS and mCIT‐C2^LACT^ fluorescence intensity ratio between outer periclinal and anticlinal PM domains in root epidermal cells after cellulase, and lysoPC/lysoPA/lysoPS treatment. Ratios have been corrected for that the anticlinal signal comes from two membranes in close proximity. Asterisks denote significant difference (***, *P* < 0.001) in the ratio of fluorescence intensity between lysoPA/lysoPS treatment and control (lysoPC). Error bars show SE of the mean (*n* = 30). Statistical analyses were carried out using Student’s *t*‐test. LysoPC, lysophosphatidylcholine; lysoPE, lysophosphatidylethanolamine; lysoPI, lysophosphatidylinositol.

Next, we tested the effect of artificially increased phospholipid levels on CIRA. WT, *cira12* and *cira13* seedlings were treated with lysophospholipids, which are taken up and rapidly converted into phospholipids (Hishikawa *et al*., 2008; Wang *et al*., 2012; Lager *et al*., 2013; Karki *et al*., 2019). Seedlings were pretreated with cellulase for 1 h and supplemented with lysophospholipids during another hour of incubation. Two anionic lysophospholipids suppressed CIRA; WT seedlings showed a significant increase in alamethicin‐dependent ion release after lysoPS treatment (171%; *P <* 0.01) and to a lesser extent after lysoPA (46%; *P <* 0.05) treatment, as compared to the untreated seedlings and seedlings treated with lysoPC, lysoPE or lysoPI (Fig. 3a). Also, it was noted that all *cira* mutants tested displayed a small yet significant increase in ion release after treatment with lysoPS and lysoPA, as compared to the controls (Fig. 3a). Likewise, in WT seedlings treated with lysoPS without cellulase, alamethicin‐induced ion release was significantly increased as compared to controls (Fig. S7b), although lysophospholipids alone did not affect ion release (Fig. S7a). We also observed that an increase in alamethicin permeability in WT seedlings with increasing lysoPS concentration and incubation time, further indicating that the inhibition of CIRA in WT seedlings depends on the uptake of lysoPS (Fig. S7c).

Confocal microscopy was performed on *Arabidopsis* seedlings after external application of lysoPS and lysoPA, using lysoPC as a control. Around a twofold increase in PS in the outer periclinal/anticlinal ratio of PM was observed after lysoPS addition to cellulase‐treated roots, as compared to the lysoPC control (Figs 3b,c, S8a), bringing the PS ratio back to noncellulase‐treated levels (Fig. 2). However, no change was observed in PA distribution in PM after lysoPA treatment (Figs 3b,c, S8a). Moreover, no difference in cytoplasm/anticlinal PM ratio was observed after lysoPS/lysoPA/lysoPC application (Fig. S8b). These results show that an increase in negatively charged lipids, especially PS, in the outer periclinal PM can negatively affect CIRA.

### CIRA depends on vesicular trafficking

**Fig. 4:**
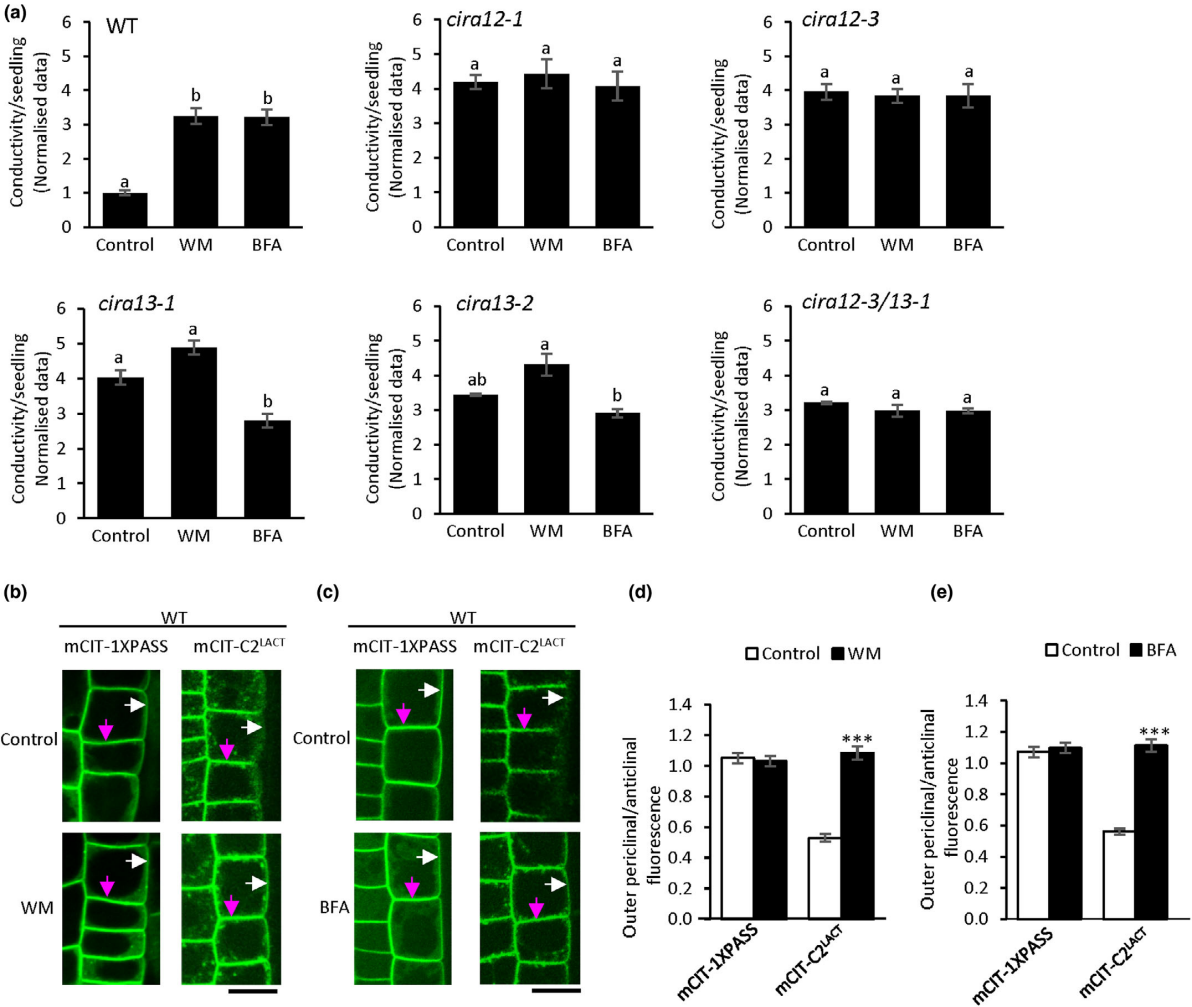
Cellulase‐induced resistance to alamethicin (CIRA) depends on membrane vesicular trafficking. (a) The inhibitors of endocytosis, wortmannin (WM), and exocytosis, brefeldin A (BFA), were tested for effects on CIRA in wild‐type (WT) and *cira* mutants. Seedlings were pretreated with BFA or WM followed by cellulase and then tested for alamethicin‐dependent ion release. Application of WM and BFA counteracted CIRA in WT. A significant increase in alamethicin‐dependent ion release was observed in c*ira13* mutants post‐WM and BFA treatment. Seedlings pre‐treated with cellulase alone were used as controls. Data were normalised by dividing by the mean of the WT control, the average of which was 0.57 ± 0.02 μS cm^−1^ per seedling (mean ± SE). Values marked by the same letter are not statistically different (*P <* 0.05). Error bars show SE of the mean (*n* = 3). Statistical analyses were carried out using one‐way analysis of variance (ANOVA) followed by Tukey *post‐hoc* analysis (denoted by letters). (b, c) Root early extension zone epidermis of WT *Arabidopsis* seedlings expressing distribution probes mCIT‐1XPASS, mCIT‐C2^LACT^ treated with cellulase and WM/BFA were analysed by spinning disc confocal microscopy. Magenta arrows highlight dual anticlinal plasma membranes (PMs) separated by a cell wall; white arrows indicate the outer periclinal PM. Bars, 10 μm. (d, e) Quantified mCIT‐1XPASS and mCIT‐C2^LACT^ fluorescence intensity ratio between outer periclinal and anticlinal PM domains in root epidermal cells after cellulase, and WM/BFA treatment. Ratios have been corrected for the fact that the anticlinal signal comes from two membranes in close proximity. Asterisks denote significant difference (***, *P* < 0.001) in the ratio of fluorescence intensity between inhibitor‐treated and control. Error bars show SE of the mean (*n* = 30). Statistical analyses were carried out using Student’s *t*‐test (denoted by asterisks).

CIRA12 and CIRA13 are located in intracellular membranes, yet the induction of alamethicin resistance is detected in the PM. PS removal from the outer periclinal PM, resulting in CIRA, may depend on endocytic PS internalization, and recycling of PS‐depleted vesicles to the PM. To test this, we monitored the effect of vesicular trafficking inhibitors by external application of the exocytosis inhibitor BFA and the endocytosis inhibitor WM. When pretreated with either of these inhibitors, the CIRA response was suppressed in WT, but *cira12* seedlings were unaffected (Fig. 4a). Interestingly, BFA‐treated *cira13‐1* (but not *cira13‐2*) seedlings displayed a significant decrease in alamethicin‐dependent ion release compared with the control (Fig. 4a). However, the *cira13‐1 and cira13‐2* mutants displayed the same differential effect, with less ion release in BFA‐ than in WM‐treated seedlings (Fig. 4a). The double mutant was unaffected, similar to *cira12* mutant lines (Fig. 4a). Hence, the opposite effects of BFA and WM observed in *cira13* require the presence of CIRA12/PSD3, as these effects are lost in the double mutant (Fig. 4a). Since both WM and BFA can affect various processes in plant cells, and consequently tend to produce quite pleiotropic effects, especially with long‐term application (Emans *et al*., 2002; Ritzenthaler *et al*., 2002; Wang *et al*., 2009), WT and mutant seedlings were also treated with tyrphostin A (T) that inhibits clathrin‐dependent endocytosis (Dhonukshe *et al*., 2007), and endosidin2 (E) that inhibits exocytosis by binding to an exocyst complex (Zhang *et al*., 2016). These compounds merely induced a partial inhibition of CIRA in WT seedlings and had no effect in *cira* mutants, indicating that clathrin‐dependent endocytosis and the exocyst complex have minor roles in CIRA (Fig. S9).

Confocal microscopy was performed on *Arabidopsis* seedlings expressing mCIT‐1xPASS and mCIT‐C2^LACT^, after external application of WM and BFA. An around twofold increase in the PS outer periclinal/anticlinal ratio of PM was observed after WM (Figs 4b,d, S10a) or BFA (Figs 4c,e, S10c) addition to cellulase‐treated roots, as compared to WM/BFA‐untreated controls (Figs 4b–e, S8a). However, no change was observed in PA distribution in PM after WM/BFA treatment (Figs 4b–e, S10a,c). Besides, no difference in cytoplasm/anticlinal PM ratio was observed after WM/BFA application (Fig. S10b,d). These results indicate that vesicle trafficking plays an important role in CIRA.

**Fig. 5:**
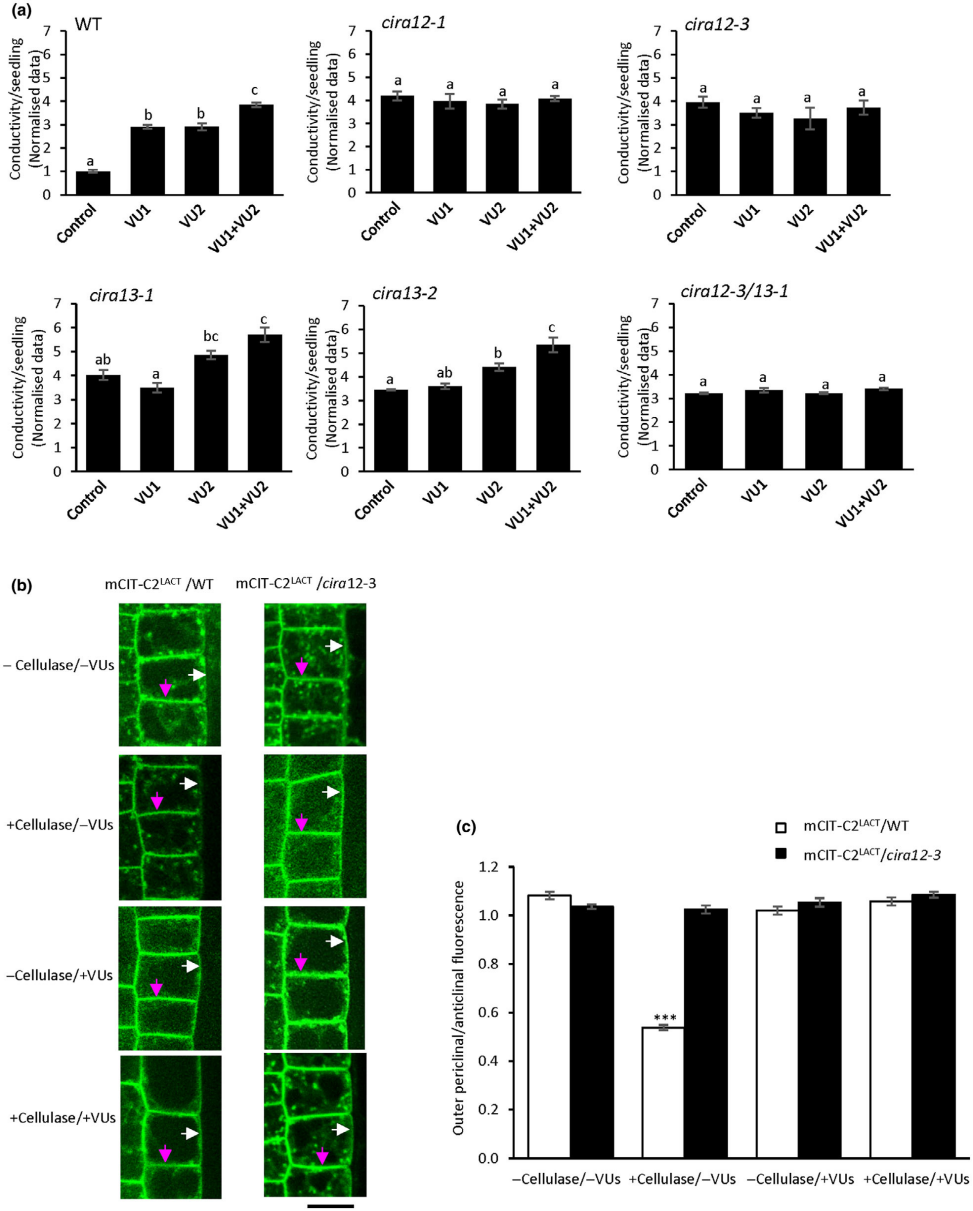
PHOSPHOLIPASE Dζ inhibitors prevent cellulase‐induced resistance to alamethicin (CIRA). (a) The effect of the inhibitors VU0359595 (VU1) and VU0285655‐1 (VU2) on CIRA was tested in wild‐type (WT), *cira12* and *cira13* mutants. WT, *cira12* and *cira13* mutants were treated overnight with the inhibitors followed by cellulase treatment and then tested for alamethicin‐dependent ion release. A significant increase in alamethicin‐dependent ion release was observed in *cira13* mutants post‐VU2 and VU1 + VU2 treatment. Seedlings pretreated with cellulase followed by alamethicin were used as controls. Data were normalised by dividing by the mean of the WT control, the average of which was 0.57 ± 0.02 μS cm^−1^ per seedling (mean ± SE). Values marked by the same letter are not statistically different (*P <* 0.05). Error bars show SE of the mean (*n* = 3). Statistical analyses were carried out using one‐way analysis of variance (ANOVA) followed by Tukey *post‐hoc* analysis (denoted by letters). (b, c) External application of *PLDζ‐*inhibitors (VUs) prevents cellulase‐induced anticlinal asymmetry in the plasma membrane (PM) of the WT. (b) Root early extension zone epidermis of *Arabidopsis* WT and mutant seedlings *cira12‐3* expressing the distribution probe mCIT‐tagged phosphatidylserine (PS) sensor (mCIT‐C2^LACT^) treated with and without cellulase and/or with and without *PLDζ‐*inhibitors were analysed by spinning disc confocal microscopy. Magenta arrows highlight dual anticlinal PMs separated by a cell wall; white arrows indicate the outer periclinal PM. Bar, 10 μm. (c) Quantified PS sensor fluorescence intensity ratio between outer periclinal and anticlinal PM domains in root epidermal cells with and without cellulase treatment and/or with and without *PLDζ‐*inhibitors. Asterisks denote significant difference (***, *P* < 0.001) in the ratio of fluorescence intensity between mCIT‐C2^LACT^/WT and mCIT‐C2^LACT^ */cira12‐3*. Ratios have been corrected for the anticlinal signal coming from two membranes in close proximity. Error bars show SE of the mean (*n* = 30). Statistical analyses were carried out using Student’s *t*‐test (denoted by asterisks).

As CIRA13/PLDζ2 and its product PA are involved in regulating vesicle trafficking (Li & Xue, 2007), we attempted to dissect the effects of the enzyme activity further in the context of CIRA. We therefore used the PLDζ inhibitors VU0359595 (VU1) and VU0285655‐1 (VU2) (Yao *et al*., 2013) that are considered specific for mammalian PLDζ1 (VU1) and PLDζ2 (VU2) (Scott *et al*., 2009). When WT, *cira12* and *cira13* mutants were pretreated with PLDζ inhibitors and cellulase, followed by alamethicin addition, the CIRA process was counteracted in WT seedlings by all treatments (Fig. 5a). However, the PLDζ inhibitors had no effect in *cira12* mutants (Fig. 5a). By contrast, *cira13* seedlings displayed a significantly increased alamethicin‐dependent ion release by VU1 + VU2 treatment, as compared to the control seedlings, with VU2 displaying an intermediate trend (Fig. 5a). Consistent results were observed when confocal microscopy was performed on WT and *cira12‐3 Arabidopsis* seedlings expressing PS‐specific sensors mCIT‐C2^LACT^ after ± cellulase and/or ± PLDζ inhibitors (VUs) treatment (Figs 5b, S11a). External application of VUs on cellulase‐treated seedlings resulted in around a twofold increase in PS in the outer periclinal/anticlinal ratio of PM, as compared to the cellulase‐treated seedlings without VUs, thus reverting the cellulase effect (Figs 5b,c, S11a). In addition, no difference in cytoplasm/anticlinal PM ratios was observed after VU treatment (Fig. S11b). Moreover, no changes in PA and PS distribution were observed in confocal microscopy on VU‐treated WT seedlings expressing mCIT‐1xPASS and *cira13‐1* expressing mCIT‐C2^LACT^, respectively (Fig. S12). These results show that inhibition or loss of PLDζ2 activity adversely affects CIRA. Thus, the inhibitors had an effect beyond the *cira13*/*pldζ2* mutation and are thus not completely specific inhibitors for PLDζ proteins only. As for BFA and WM treatments, the *cira12/cira13* seedlings were similar to the *cira12* mutants; that is the effect of the presence or absence of PLDζ and vesicular transport activity depends on the presence of CIRA12, which is epistatic to CIRA13 in the presence of the inhibitors. Taken together, these results suggest that CIRA13/PLDζ2‐mediated activation of vesicular transport is required for CIRA.

## Discussion

Symbiotic fungi in the genus *Trichoderma* secrete membrane‐permeabilising antimicrobial peptaibols such as alamethicin as part of mycoparasitism and can in that way protect the host plant from pathogens (Druzhinina *et al*., 2011). While alamethicin lyses cells of invading pathogens, it can also lead to host plant cell lysis and cell death (Rippa *et al*., 2010; Dotson *et al*., 2018). However, upon pretreatment of plant cells with cellulase from *T. viride*, the CIRA process prevents the alamethicin‐induced permeabilization (Aidemark *et al*., 2010; Dotson *et al*., 2018). In *Arabidopsis*, CIRA was identified in primary root epidermal cells (Dotson *et al*., 2018). This study further demonstrates that CIRA is active also in lateral roots irrespective of nutrient conditions and we identified two genes that are involved in the CIRA process under all tested conditions (Fig. 1).

An altered anionic lipid composition of the PM was found in conjunction with CIRA in cultured tobacco cells (Aidemark *et al*., 2010). The molecular backbone of all phospholipids is the anionic PA moiety, which is a phospholipid by itself and, by modifications to the phosphate group can be converted into the zwitterionic phospholipids PC and PE, or the anionic phospholipids PS and PI (Uemura *et al*., 1995; Colin & Jaillais, 2020). PI is then stepwise phosphorylated into anionic PI4P and PI(4,5)P_2_, which can accumulate in the PM (Simon *et al*., 2016; Dubois & Jaillais, 2021). PS can be converted to PE by CIRA12/PSD3 in the ER, and this study reveals that its activity is crucial for CIRA (Fig. 1a–d). Similarly, the enzyme PLDζ2, which can produce PA from PC in the tonoplast, TGN and MVBs, is also essential for the CIRA process (Fig. 1a–d). Genes encoding the enzymes that catalyse the production (*PLDζ2/CIRA13*) as well as the conversion (*PSD3/CIRA12*) of anionic phospholipids are to the best of our knowledge, the first host genes identified to be essential for a specific *Trichoderma*–plant interaction. Although *Trichoderma*‐induced upregulation of biotic and abiotic stress‐related genes and proteins has been reported in plants, including *Arabidopsis* (Morán‐Diez *et al*., 2012; Brotman *et al*., 2013), maize (Shoresh & Harman, 2008) and oil palm (Ho *et al*., 2016), none have been confirmed essential during *Trichoderma* root colonisation or *Trichoderma*–plant–pathogen interaction.

The hydrophobic nature of alamethicin allows it to permeabilise cells by inserting itself into cell membranes and forming pores (Jen *et al*., 1987). In artificial membranes, alamethicin forms pores only when applied from the net positively charged side of a membrane, which has a transmembrane potential (Duclohier & Wroblewski, 2001). Such transmembrane potential‐dependent permeabilization was also observed in tobacco cells, in which the PM with negative transmembrane potential is permeabilised by alamethicin, but not the tonoplast that has a positive transmembrane potential (Matic *et al*., 2005). The alamethicin pore formation depends on not only the transmembrane potential but also the lipid:peptide ratio, and peptide and ion concentration (Bezrukov *et al*., 1998; Aguilella & Bezrukov, 2001; Duclohier & Wroblewski, 2001; Thippeswamy *et al*., 2009). The alamethicin concentration required for permeabilization can also be altered by artificial or genetic changes in the lipid headgroups that modify the average surface charge of the membrane (Heller *et al*., 1997; Thippeswamy *et al*., 2009). The CIRA‐negative phenotype of *psd3/cira12* (Fig. 1c,d) suggests that a decrease in PS in the PM makes the membrane resistant to alamethicin, likely commencing via a decrease in the membrane surface charge, secondarily resulting in increased lipid packing. Consistent with the current results, decreased PS and PI levels were observed in the cellulase‐treated cultured tobacco cells that were resistant to alamethicin permeabilization (Aidemark *et al*., 2010). Correspondingly, external application of lysoPS strongly counteracted CIRA in WT seedlings (Fig. 3a). LysoPS is rapidly converted to PS (Hishikawa *et al*., 2008) in the PM, making the membrane surface more anionic, which would result in loosened lipid packing and lower alamethicin resistance. Consistently, fungal cells, which are the natural targets of peptaibols, generally have a highly negative PM surface potential (Rautenbach *et al*., 2016), and a pH‐induced more negative surface potential of artificial zwitterionic membranes was observed to increase the pore formation of alamethicin (Chiriac & Luchian, 2007). The addition of lysoPA also reduced resistance to alamethicin permeabilization in WT seedlings, but to a substantially lesser extent than lysoPS did (Fig. 3a). It is known that anionic phospholipids (PS, PA and PI4P) are the lipids mainly responsible for the surface charge of the PM (Noack & Jaillais, 2020). The relative contributions of the specific phospholipids to the total membrane surface charge are not known for multicellular eukaryotes, whereas PS is the major anionic phospholipid responsible for PM surface potential in yeast (Moravcevic *et al*., 2010). Consistent with the *cira12* phenotype, the significant decrease in CIRA after lysoPS treatment indicates that PS is a crucial factor for PM surface charge and alamethicin permeabilization in *Arabidopsis* root epidermis.

Alamethicin can form pores in membranes only when applied from the net positive side; hence, external alamethicin can permeabilise the PM and permeate into the cell but cannot permeabilise the PM from the cytosolic side (Duclohier & Wroblewski, 2001; Matic *et al*., 2005; Dotson *et al*., 2018). So, in root epidermis, alamethicin will mainly affect the outer periclinal PM in which it can lead to pore formation and membrane permeabilization. The addition of cellulase resulted in decreased levels of PS specifically in the outer periclinal PM but not in the anticlinal PM, and induced a consistent reduction in negative surface charge in the outer periclinal PM (Fig. 2). The decrease in PS in the outer periclinal PM was also reversed with external application of lysoPS (Fig. 3b,c). Due to the internal location of the fluorescent PS probe, these detected changes all take place in the inner leaflet of the PM. In mammals, the PM distribution of PS is transversally asymmetric with PS mainly found in the inner leaflet and zwitterionic phospholipids such as PC preferentially located in the outer leaflet (Hale *et al*., 1996). However, during programmed cell death, PS moves from the inner to the outer leaflet by scramblase action (Nagata, 2018). In the fungus *Fusarium oxysporum*, apoptosis induced by the peptaibol Trichokonin VI also occurs concomitantly with exposure of PS at the external surface of the cell membrane (Shi *et al*., 2012). In plant PM, a transversal asymmetry of PS, being absent in the outer leaflet yet exposed upon cell death, has been reported (O'brien *et al*., 1998), but only in protoplasts, which have had their cell walls removed using the standard *Trichoderma* cellulase. The choice of protoplasts as material was likely because the cell wall hinders access for the common PS reporter annexin V. However, since we here show that PS becomes depleted in the PM upon treatment with cellulase (Fig. 2), we must conclude that it is presently not known whether there is a PS asymmetry in the plant PM under normal conditions. Regarding alamethicin sensitivity, the transversal asymmetry may be less important because several maAMPs, including alamethicin, induce flip‐flop of anionic phospholipids when binding to the surface of membrane vesicles; alamethicin disrupts lipid packing in the PM resulting in the loss of lipid asymmetry (Qian & Heller, 2011; Qian *et al*., 2014; Rai *et al*., 2016; Nguyen *et al*., 2019; Taylor *et al*., 2019). Furthermore, already a change of the inner leaflet surface charge will modify the lipid packing and as a consequence the insertion of alamethicin into the membrane (Aguilella & Bezrukov, 2001).

Mammalian epithelial cell PMs are partitioned laterally into outer periclinal, anticlinal and basal domains with different functionalities, corresponding to the outer periclinal, anticlinal and basal PM of the root epidermis cells. Less is known about lateral asymmetry in plant PM, that is the unequal distribution of lipids between the outer periclinal and the anticlinal and basal PM regions, yet domains enclosing proteins such as auxin‐interacting factors, GTPases and CASPARIAN STRIP MEMBRANE PROTEINs have been localised to different PM regions of particular cell layers (Galweiler *et al*., 1998; Geldner, 2013; Malinsky *et al*., 2013; Cassim *et al*., 2019). Among lipids, particular sterols, PIs and DAG have been localised to the outer periclinal PM of growing pollen tubes (Moscatelli *et al*., 2015). However, a differential lipid distribution between the outer periclinal and anticlinal PM domains of a dermal cell layer has not been observed, such as it is seen in the induced distribution of surface charge and anionic lipid changes in cellulase‐treated roots (Fig. 2). What barriers must prevent the anionic lipids from laterally diffusing between the PM domains is presently not known and a topic for further studies.

**Fig. 6:**
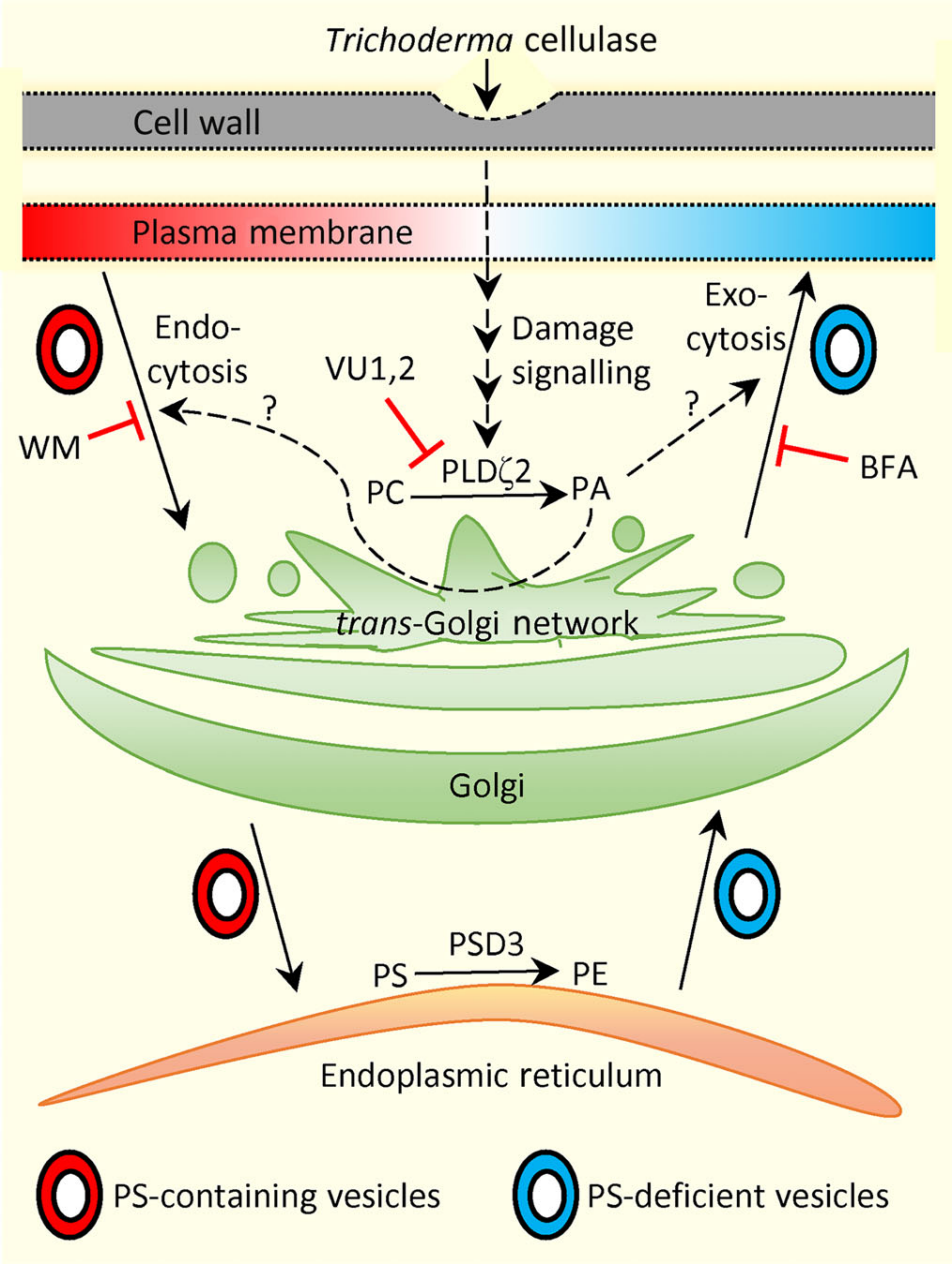
Proposed model for cellulase‐induced resistance to alamethicin (CIRA) and its inhibitor sensitivity in root epidermis. Cellulase treatment causes endocytosis of the anionic lipid phosphatidylserine (PS), which reaches the endoplasmic reticulum (ER) via vesicular trafficking activated by PLDζ2‐derived phosphatidic acid (PA) in the *trans*‐Golgi network and Golgi, although it is not known if endo‐ or exocytosis is activated by Golgi PA. PS gets converted to phosphatidylethanolamine (PE) via PS decarboxylase 3 (PSD3) in the ER. Vesicles again trafficked to the plasma membrane (PM) via exocytosis are depleted in PS. The slow depletion of the PM results in CIRA. External application of wortmannin (WM) and brefeldin A (BFA) or PLDζ inhibitors (VU1,2) blocks vesicular trafficking, preventing alamethicin resistance induction. Solid arrows denote experimentally verified processes. Blunt‐ended arrows indicate inhibition and dashed arrows indicate tentative signal processes.

CIRA12/PSD3 is localised in the ER (Nerlich *et al*., 2007), and therefore, vesicular transport is required for this enzyme's modification of the PS/PE ratio to reach the PM. The significant increase in alamethicin‐dependent ion release from cellulase‐pretreated WT seedlings post inhibition of exocytosis or endocytosis demonstrates the pivotal role of vesicle trafficking for CIRA efficacy (Fig. 4). A model is illustrated in Fig. 6. CIRA13/PLDζ2 is localised in the TGN, MVB and the tonoplast (Yamaryo *et al*., 2008; Shimamura *et al*., 2022). The mCIT‐1xPASS sensor is a PA‐specific lipid distribution sensor, but determining *cira13*‐induced changes in total PA is difficult because there are 11 other PLD enzymes and also other enzymes that can produce PA.

The different phenotypes seen in *pld* mutants indicate that, in *Arabidopsis*, the functions of the 12 PLDs are specific and not redundant (Wang, 2005; Li *et al*., 2009; Hong *et al*., 2016). While PH/PX‐PLDs are clearly structurally different from most other plant PLDs, the two PH/PX‐PLDs (PLDζ1 and PLDζ2) also differ from each other in various aspects. PLDζ1 and PLDζ2 in *Arabidopsis* are highly expressed in roots (Li *et al*., 2006a), yet phenotypes strongly deviate between the *pldζ1* and *pldζ2* mutants, indicating different physiological functions (Li *et al*., 2006a; Shi *et al*., 2012). In *Arabidopsis* under hypoxic stress, PLDζ1 and PLDζ2 activate different signalling pathways. Under such hypoxia, total protoplast PA levels were increased in WT and *pldζ1* mutants but were unchanged in *pldζ2* mutants, indicating that PLDζ2 is involved in PA production through Ca^2+^ signalling, while PLDζ1 plays a more significant role in ROS signalling (Lindberg *et al*., 2018). Likewise, *pldζ1* and *pldζ2* mutants displayed different root responses to salt stress (Ben Othman *et al*., 2017), indicating that although they produce the same compound, they are involved in distinct physiological functions within *Arabidopsis*.

The specific effects of the PLDζ2 mutation and inhibitors observed in this study and by Li *et al*. (2006a, 2006b) may be caused by changes in a particular pool of PA, which can be by association of a PLD to a particular membrane or even subdomain of a membrane. Such pool behaviour has for example been observed for PLDζ2 in response to variations in phosphate nutrition (Yamaryo *et al*., 2008; Takáč *et al*., 2019). The observation that PLDζ2 is essential for CIRA (Fig. 1), although added lysoPA poses a mildly inhibitory PLDζ2‐independent action on the CIRA process (Fig. 3), indicates that a specific pool of PA is involved in activating the CIRA process, which is in line with the need for specificity to explain mutant phenotypes for the different PLD enzymes in Arabidopsis.

PLDs and their product PA are vital in regulating vesicular transport in mammalian cells (Liscovitch *et al*., 2000; Freyberg *et al*., 2003; Jenkins & Frohman, 2005). For example, when synthesis of PA was inhibited by treating rat cells with a PLD inhibitor, it led to inhibition of vesicle secretion (Siddhanta *et al*., 2000). PLDs and PA also regulate secretion of vesicles in plant cells (Monteiro *et al*., 2005; Li & Xue, 2007). Observations by fluorescence microscopy of plant cells treated with the membrane‐selective dye FM4‐64 showed suppressed vesicle trafficking under PLDζ2 deficiency, and consistently, vesicle trafficking was enhanced by PA‐treatment or overexpression of PLDζ2 in *Arabidopsis* seedlings (Li & Xue, 2007). In this study, *pldζ2/cira13* mutants are CIRA‐negative (Fig. 1c,d) and application of PLDζ inhibitors counteracted CIRA in WT seedlings (Fig. 5), suggesting that PLDζ2 is essential for CIRA. In mammals, the PLDζ inhibitors VU1 and VU2 are specific to PLD1 and PLD2, respectively (Scott *et al*., 2009), both being of the same PLD‐types as plant PLDζ1 and PLDζ2 (Yao *et al*., 2013). However, results from this study reveal that VU1 and VU2 are not specific to either PLDζ1 or PLDζ2 in *Arabidopsis* (Fig. 5a). This harmonises with VU2 being quantitatively more efficient than VU1 in inhibiting the activity of plant PLDζ1 (Yao *et al*., 2013). Given that PLDζ2 is essential for vesicular transport and that inhibition of PLDζ2 counteracted CIRA in WT seedlings, we conclude that vesicle trafficking is essential for CIRA. Application of PLDζ inhibitors significantly increased alamethicin permeabilization in *cira13* seedlings, while no difference was observed in *cira12* and *cira12/cira13* seedlings ± PLDζ inhibitors (Fig. 5). This indicates that CIRA13/PLDζ2 is in the same pathway as CIRA12/PSD3 and that CIRA13 effects on the CIRA depend on the presence of CIRA12/PSD3, likely being a factor for communicating the lipid changes commenced by the CIRA12/PSD3‐activity to the PM. However, the upstream signalling events leading to the activation of *CIRA13/PLDζ2* are presently unknown. This report opens for future research to identify a receptor and mediators of the cellulase action.

In addition to modulating in PS and PA levels in the PM, vesicle trafficking may mediate additional changes in PM and/or cell wall composition; however, alamethicin efficacy should be independent of primary cell wall composition, mainly due to the fact that the pore size of plant cell walls of 35–52 Angstroms (Carpita *et al*., 1979) is much larger than the radius of an alpha helical 20‐mer peptide such as alamethicin (*c*. 13 Angstroms). Furthermore, the negative charge of alamethicin should hinder its binding to the cell wall. Finally, tobacco protoplasts, devoid of cell wall, were resistant to alamethicin, as were cells that had been through a mild cellulase treatment (Aidemark *et al*., 2010).

Taken together, this study shows that particular plant genes are essential for a molecular process involved in the symbiotic plant–Trichoderma interaction and being a unique induced resistance against a membrane‐active antibiotic peptide. It suggests that upon cellulase treatment, PS in the outer periclinal PM is transported via endocytosis to the ER, in which PSD3 catalyses the conversion of PS to PE, which in turn is recycled to the PM via exocytosis. The vesicular transport of PS and PE requires activation by PLDζ2, which is located in the Golgi, TGN or MVBs. Hence, a laterally asymmetric loss of anionic phospholipids in the PM prevents alamethicin permeabilization, most likely by enhancing lipid packing resulting in CIRA.

## Competing interests

None declared.

## Author contributions

AGR and BRD conceived and designed the research project. SPN, SH, SR and BRD performed the experiments. SPN, BRD, LN, PD, SW, SP, IL and AGR analysed the data. SPN and AGR wrote the manuscript, and all authors read and approved the final version of the manuscript.

## Disclaimer

The New Phytologist Foundation remains neutral with regard to jurisdictional claims in maps and in any institutional affiliations.

## Supporting information


**Fig. S1** Positive identification of *cira12* and *cira13* mutant lines.
**Fig. S2** The effect of alamethicin on ion release to the medium in wild‐type and *cira12‐1* seedlings treated ± cellulase.
**Fig. S3** Analysis of *pldz1*, *psd2*, *pss1* and *rop* mutant lines.
**Fig. S4** Inhibitors of synthesis of DAG kinase‐derived phosphatidic acid (PA) and PI4P does not affect cellulase‐induced resistance to alamethicin (CIRA) in wild‐type seedlings but display minor CIRA‐promoting effects in CIRA‐mutants.
**Fig. S5** Cellulase‐induced lateral asymmetry is observed in the plasma membrane of the wild‐type; additional information.
**Fig. S6** Cellulase‐induced lateral asymmetry in the plasma membrane is not observed in *cira* mutants; additional information.
**Fig. S7** External application of lysophospholipids alone does not affect ionic release in wild‐type seedlings, but lysophosphatidylserine (lysoPS) enhances the basal alamethicin effect.
**Fig. S8** External application of anionic phospholipid lysophosphatidylserine (lysoPS) counteracts cellulase‐induced resistance to alamethicin (CIRA) in wild‐type; additional information.
**Fig. S9** Clathrin‐dependent endocytosis and exocyst complex has a minor importance on cellulase‐induced resistance to alamethicin (CIRA).
**Fig. S10** Cellulase‐induced resistance to alamethicin (CIRA) depends on membrane vesicular trafficking; additional information.
**Fig. S11** PHOSPHOLIPASE Dζ inhibitors prevent cellulase‐induced resistance to alamethicin (CIRA) in wild‐type (WT) but not in *cira12*; additional information.
**Fig. S12** PHOSPHOLIPASE Dζ inhibitors do not affect *cira13*; additional information.
**Table S1** PCR primers used in this study.
Please note: Wiley is not responsible for the content or functionality of any Supporting Information supplied by the authors. Any queries (other than missing material) should be directed to the *New Phytologist* Central Office.

## Data Availability

The data that support the findings of this study are available within the article and/or its Supporting Information (Figs S1–S12; Table S1).
