# Supplementary material for: Arabidopsis phospholipid modifications mediate cellulase‐induced resistance to a fungal peptide antibiotic by imposing cell polarity
Source: New Phytol. 2025 Nov 8;249(2):975–91. doi: 10.1111/nph.70721 (PMC12712430; doi:10.1111/nph.70721)
Supplement: Supplementary file 1 — Fig. S1 Positive identification of cira12 and cira13 mutant lines. Fig. S2 The effect of alamethicin on ion release to the medium in wild‐type and cira12‐1 seedlings treated ± cellulase. Fig. S3 Analysis of pldz1, psd2, pss1 and rop mutant lines. Fig. S4 Inhibitors of synthesis of DAG kinase‐derived phosphatidic acid (PA) and PI4P does not affect cellulase‐induced resistance to alamethicin (CIRA) in wild‐type seedlings but display minor CIRA‐promoting effects in CIRA‐mutants. Fig. S5 Cellulase‐induced lateral asymmetry is observed in the plasma membrane of the wild‐type; additional information. Fig. S6 Cellulase‐induced lateral asymmetry in the plasma membrane is not observed in cira mutants; additional information. Fig. S7 External application of lysophospholipids alone does not affect ionic release in wild‐type seedlings, but lysophosphatidylserine (lysoPS) enhances the basal alamethicin effect. Fig. S8 External application of anionic phospholipid lysophosphatidylserine (lysoPS) counteracts cellulase‐induced resistance to alamethicin (CIRA) in wild‐type; additional information. Fig. S9 Clathrin‐dependent endocytosis and exocyst complex has a minor importance on cellulase‐induced resistance to alamethicin (CIRA). Fig. S10 Cellulase‐induced resistance to alamethicin (CIRA) depends on membrane vesicular trafficking; additional information. Fig. S11 PHOSPHOLIPASE Dζ inhibitors prevent cellulase‐induced resistance to alamethicin (CIRA) in wild‐type (WT) but not in cira12; additional information. Fig. S12 PHOSPHOLIPASE Dζ inhibitors do not affect cira13; additional information. Table S1 PCR primers used in this study. Please note: Wiley is not responsible for the content or functionality of any Supporting Information supplied by the authors. Any queries (other than missing material) should be directed to the New Phytologist Central Office. [file NPH-249-975-s001.pdf]

New Phytologist Supporting Information

Article title: ***Arabidopsis* phospholipid modifications mediate cellulase-induced resistance to a fungal peptide antibiotic by imposing cell polarity**

Authors: Saritha Panthapulakkal Narayanan, Bradley R. Dotson, Lise Noack, Sanjana Holla, Shichao Ren, Peter Dörmann, Susanne Widell, Staffan Persson, Ida Lager and Allan G. Rasmusson

Article acceptance date: 15 October 2025

The following Supporting Information is available for this article:

Fig. S1 Positive identification of *cira12* and *cira13* mutant lines.

Fig. S2 The effect of alamethicin on ion release to the medium in wild type (WT) and *cira12-1* seedlings treated ± cellulase.

Fig. S3 Analysis of *pldz1, psd2, pss1* and *rop* mutant lines.

Fig. S4 Inhibitors of synthesis of DAG kinase-derived phosphatidic acid (PA) and PI4P does not affect cellulase-induced resistance to alamethicin (CIRA) in wild type seedlings but display minor CIRA-promoting effects in CIRA-mutants.

Fig. S5 Cellulase-induced lateral asymmetry is observed in the plasma membrane of the wild-type; additional information.

Fig. S6 Cellulase-induced lateral asymmetry in the plasma membrane is not observed in *cira* mutants; additional information.

Fig. S7 External application of lysophospholipids alone does not affect ionic release in wild type seedlings, but lysophosphatidylserine (lysoPS) enhances the basal alamethicin effect.

Fig. S8 External application of anionic phospholipid lysophosphatidylserine (lysoPS) counteracts cellulase-induced resistance to alamethicin (CIRA) in wild type; additional information.

Fig. S9 Clathrin dependent endocytosis and exocyst complex has a minor importance on cellulase-induced resistance to alamethicin (CIRA).

Fig. S10 Cellulase-induced resistance to alamethicin (CIRA) depends on membrane vesicular trafficking; additional information.

Fig. S11 PHOSPHOLIPASE Dζ inhibitors prevent cellulase-induced resistance to alamethicin (CIRA) in WT but not in *cira12*; additional information.

Fig. S12 PHOSPHOLIPASE Dζ inhibitors do not affect *cira13*; additional information.

Table S1 PCR primers used in this study.

Fig. S1 Positive identification of *cira12* and *cira13* mutant lines. Upper panel, PCR genotyping on genomic DNA from leaves of *Arabidopsis* wild type (WT) and *cira* mutants. Lower panel, RT-PCR analysis using RNA isolated from leaves of WT and *cira* mutants. Primers used for genotyping and RT-PCR are shown in Table S1.

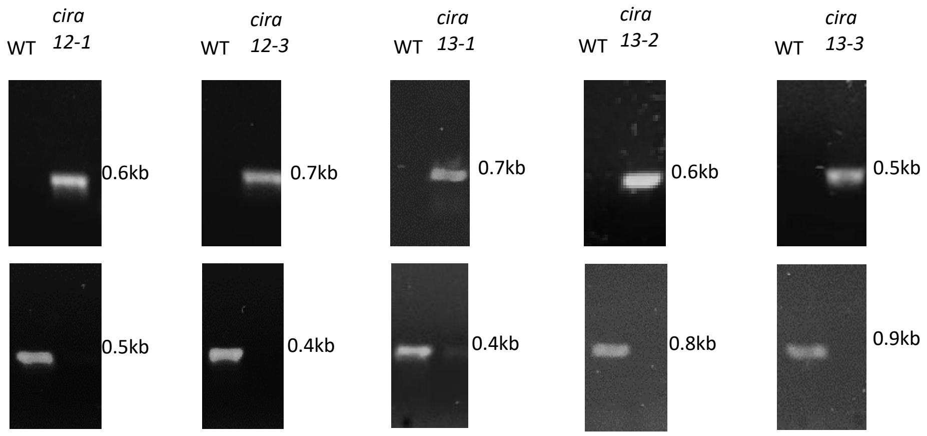

Fig. S2 The effect of alamethicin on ion release to the medium in wild type (WT) and *cira12-1* seedlings treated ± cellulase. Alamethicin-dependent ion release was measured as conductivity after treating WT and *cira12-1* seedlings at different concentrations of alamethicin without cellulase **(a)** and after pretreatment with 1% (w/v) cellulase for 2 h **(b).**

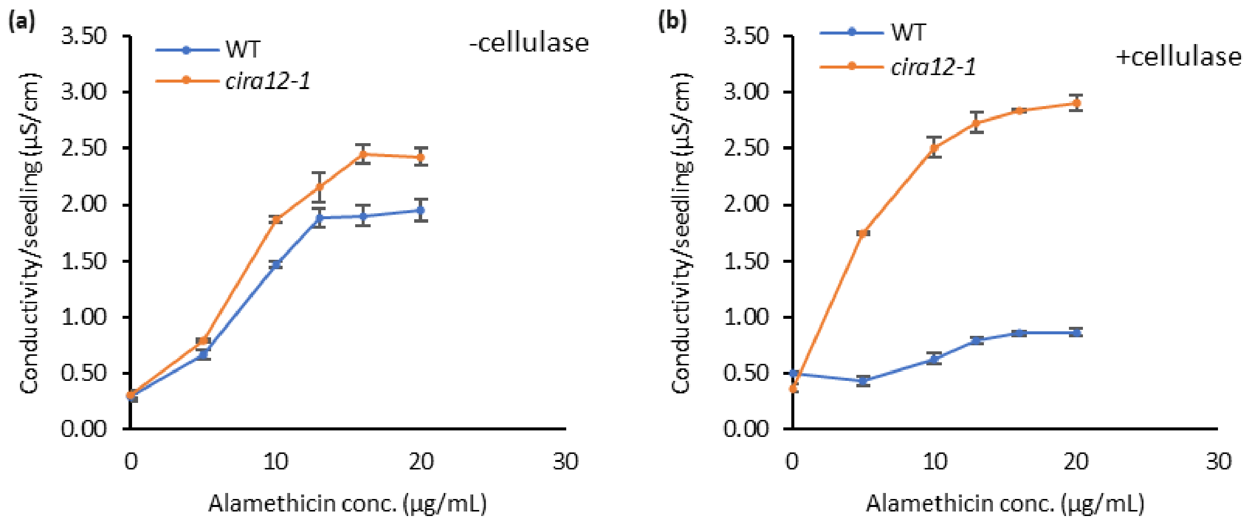

Fig. S3 Analysis of *pldz1, psd2, pss1* and *rop* mutant lines. Schematic map of the T-DNA insertions **(a)** *PLDZ1* (At3g16785), **(b)** *PSD2* (At5g57190) and (**c**) *PSS1* (At1g15110) genomic sequences (scale=150 bp). White boxes represent exons. LBb1.3, SALK T-DNA border primer; LP, left primer; RP, right primer; LBGABI, GABI T-DNA border primer. **(d)** PCR genotyping on genomic DNA from leaves of *Arabidopsis* wild type (WT), *pldz1, psd2* and *pss1* mutants. **(e)** Alamethicin-dependent ion release in WT, *pldz1* and *psd2* seedlings. Asterisks denote significant difference (**, $p < 0.01$) in ion conductivity in samples post cellulase pre-treatment (C+A) as compared to the samples without cellulase pretreatment (A). Error bars show standard error of the mean (n=3). (**f**) Since *pss1* homozygous lines were sterile and had to be propagated as heterozygotes, fluorescent microscopy was performed to detect cellulase-induced resistance to alamethicin (CIRA). Representative images of primary roots of WT and the *pss1* mutant showing CIRA was taken using a fluorescence microscope equipped with a G2A filter. (**g**) Images of primary roots of WT and *rop* mutants showing CIRA taken using the fluorescence microscope. Seedlings were grown vertically in ½ MS-Agar for 14 days **(f)** or 7 days **(g)** and incubated in cellulase (C) for 2 h, and then for 10 min with 10 µg/ml alamethicin (A), where PrI was included during the last min. Bar correspond to 100 µm. The images show one representative replicate out of five. BF, bright field.

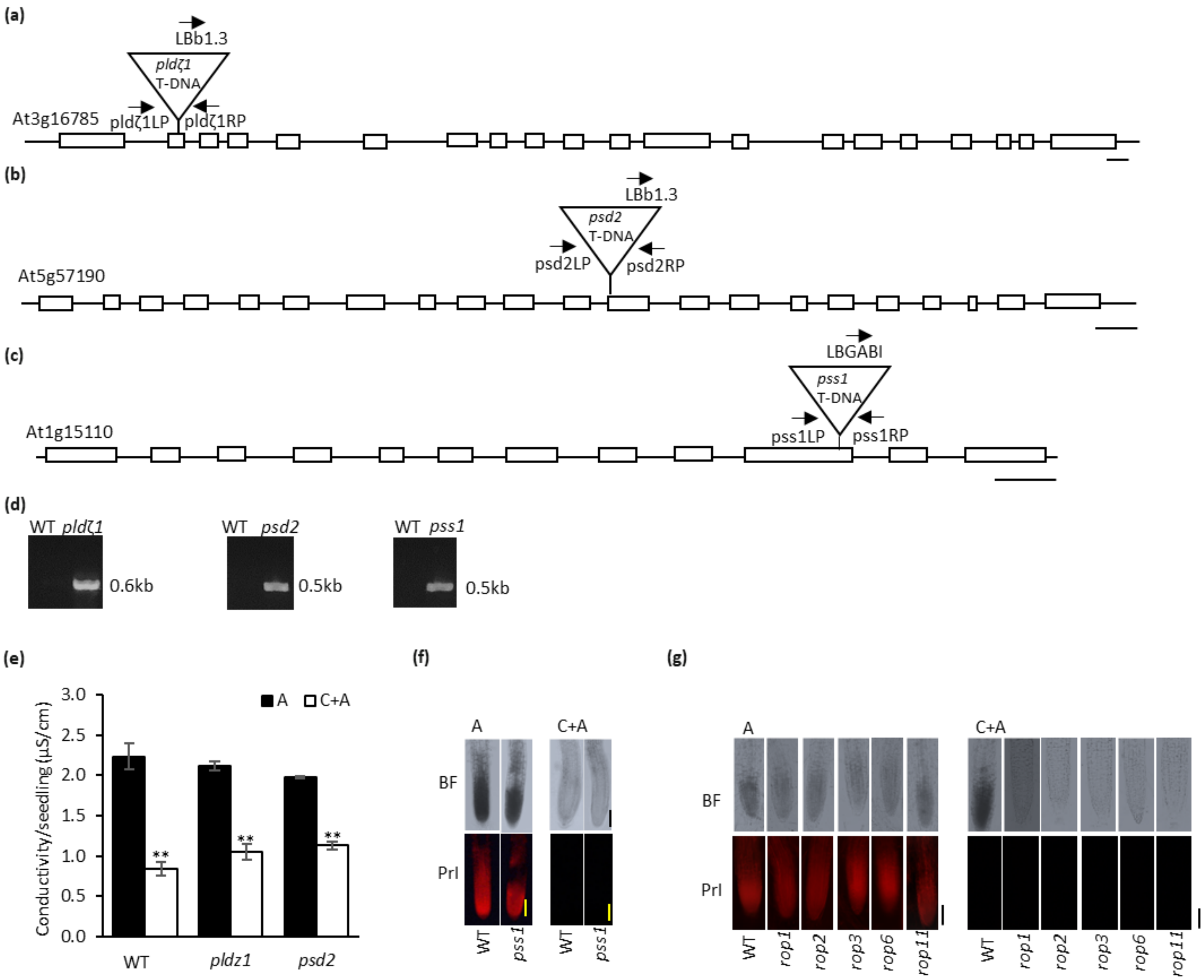
(a)
LBb1.3
pldζ1
T-DNA
At3g16785
pldζ1LP
pldζ1RP
(b)
LBb1.3
psd2
T-DNA
psd2LP
psd2RP
At5g57190
(c)
LBGABI
pss1
T-DNA
At1g15110
pss1LP
pss1RP
(d)
WT pldζ1
0.6kb
WT psd2
0.5kb
WT pss1
0.5kb
(e)
A
C+A
Conductivity/seedling (µS/cm)
3.0
2.5
2.0
1.5
1.0
0.5
0.0
**
**
**
WT
pldz1
psd2
(f)
A
C+A
BF
PrI
WT
pss1
WT
pss1
(g)
A
C+A
BF
PrI
WT
rop1
rop2
rop3
rop6
rop11
WT
rop1
rop2
rop3
rop6
rop11

Fig. S4 Inhibitors of synthesis of DAG kinase-derived phosphatidic acid (PA) and PI4P does not affect cellulase-induced resistance to alamethicin (CIRA) in wild type seedlings but display minor CIRA-promoting effects in CIRA-mutants. The effect of DAG kinase inhibitor (R59949 (R)) and PI4P formation inhibitor (PAO) on CIRA was tested in wild type and mutants. (**a**), Seedlings were treated with the inhibitors for 60 min, washed twice in water followed by cellulase (C) for 2 h, washed thrice in water and then tested for alamethicin (A)-dependent ion release. (**b**), Seedlings treated with inhibitors and cellulase and tested for alamethicin-independent ion release. Seedlings treated without inhibitors were used as controls. Seedlings were grown in ½ MS for 6 days prior to treatments. Values marked by the same letter are not statistically different ($p < 0.01$). Error bars show standard error of the mean (n=3).

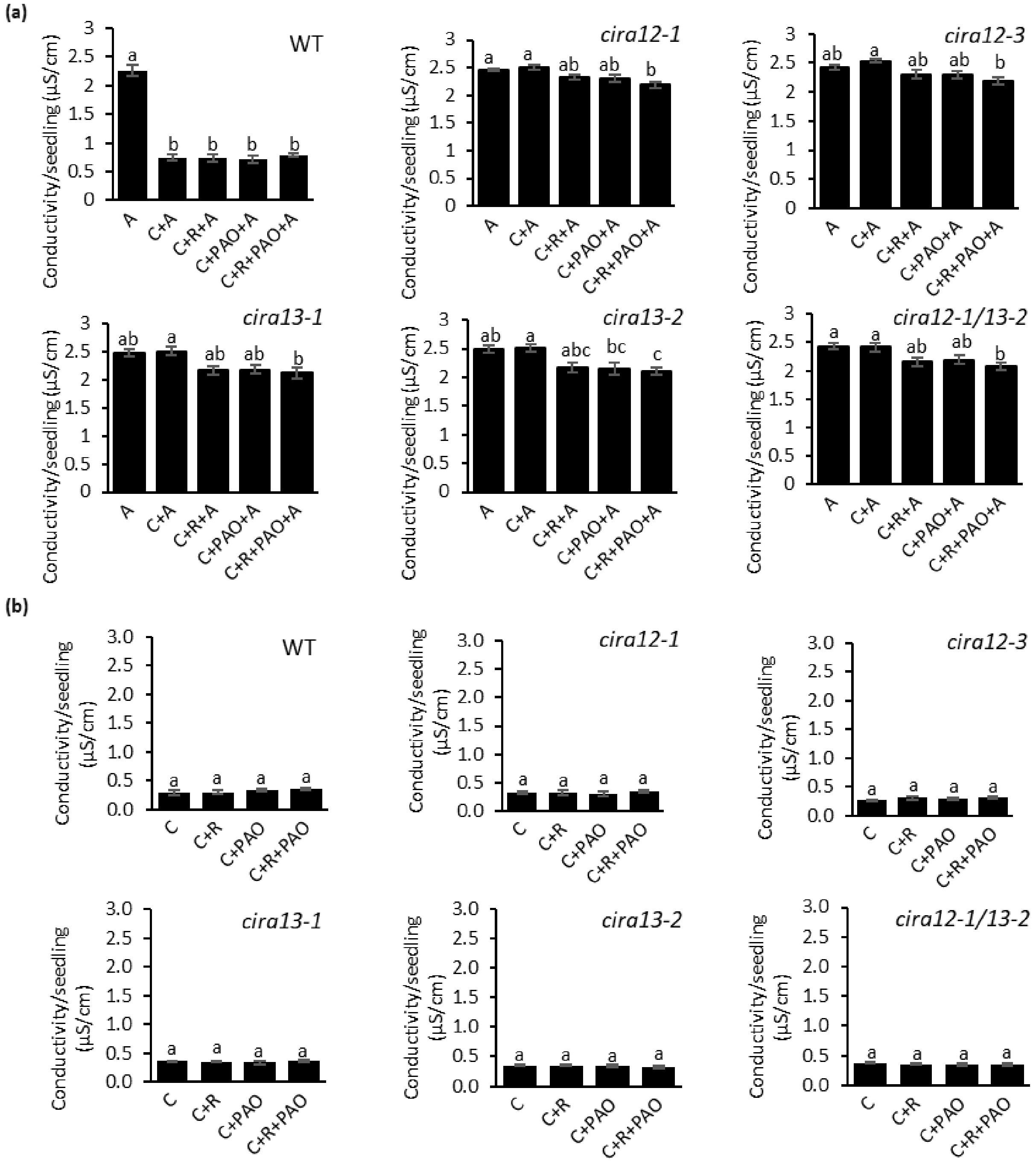
(a)
WT
cira12-1
cira12-3
cira13-1
cira13-2
cira12-1/13-2
Conductivity/seedling (µS/cm)
A
C+A
C+R+A
C+PAO+A
C+R+PAO+A
(b)
WT
cira12-1
cira12-3
cira13-1
cira13-2
cira12-1/13-2
Conductivity/seedling (µS/cm)
C
C+R
C+PAO
C+R+PAO

Fig. S5 Cellulase-induced lateral asymmetry is observed in the plasma membrane of the wild-type; additional information. (a) Root early extension zone epidermis of triple replicate seedlings of WT *Arabidopsis* expressing distribution probes mCIT-1XPASS, mCIT-C2^{LACT} and mCIT-KA1^{MARK1} treated 2 h with and without cellulase were analyzed by spinning disc confocal microscopy. Magenta arrows highlight dual anticlinal plasma membranes (PMs) separated by a cell wall; white arrows indicate outer periclinal single PM. Scale bar, 10 µm. **(b)** Quantified phosphatidic acid (PA), phosphatidylserine (PS) and charge sensor fluorescence intensity ratio between cytoplasm and anticlinal PM domains in root epidermal cells with and without cellulase treatment. Error bars show standard error of the mean (n=30). Error bars show standard error of the mean (n=30).

(a)

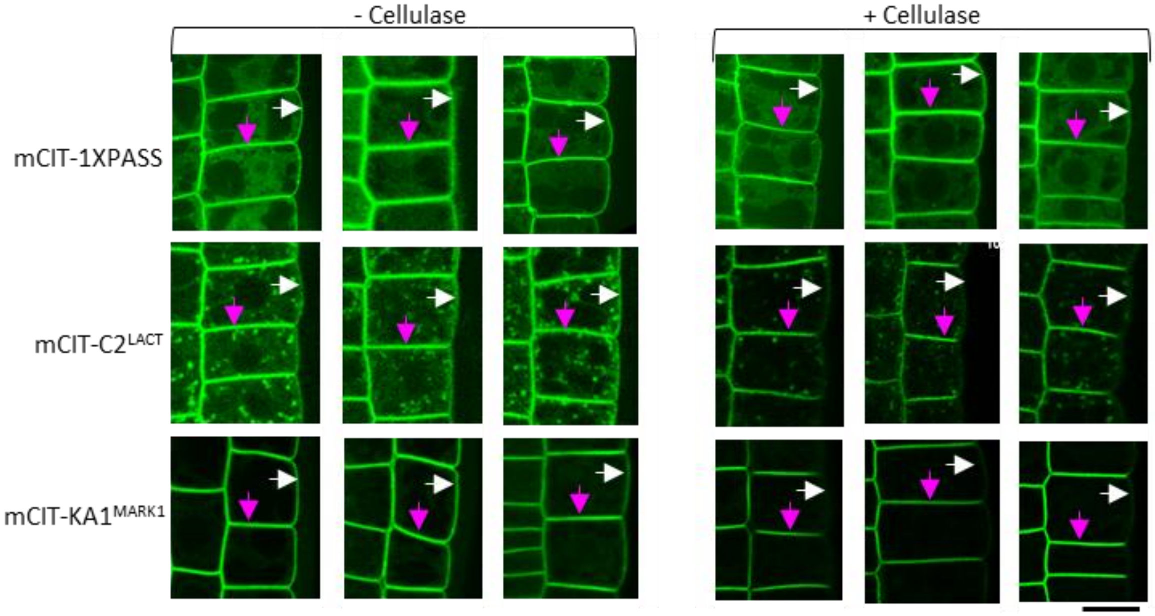
- Cellulase
+ Cellulase
mCIT-1XPASS
mCIT-C2LACT
mCIT-KA1MARK1

(b)

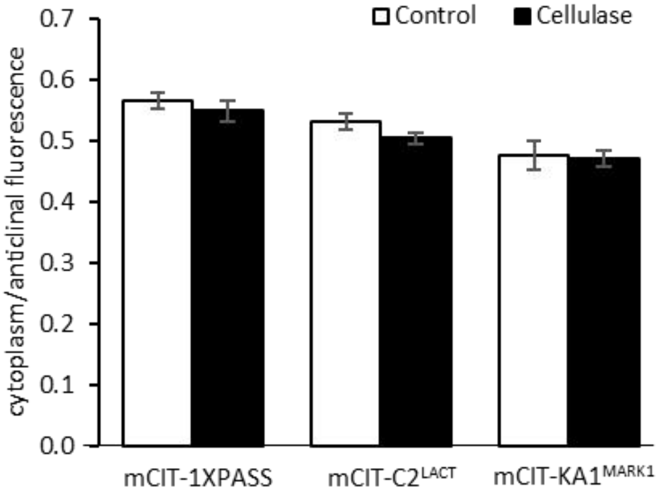
Control
Cellulase
cytoplasm/anticlinal fluorescence
0.7
0.6
0.5
0.4
0.3
0.2
0.1
0.0
mCIT-1XPASS
mCIT-C2LACT
mCIT-KA1MARK1

Fig. S6 Cellulase-induced lateral asymmetry in the plasma membrane is not observed in *cira* mutants; additional information. **(a**) Root early extension zone epidermis of *Arabidopsis* mutant seedlings *cira13-1* expressing distribution probes mCIT-1XPASS, mCIT-C2^{LACT}, mCIT-KA1^{MARK1} and *cira12-3* expressing mCIT-C2^{LACT}, mCIT-KA1^{MARK1} were treated 2 h with and without 1 % cellulase and analyzed by spinning disc confocal microscopy. Magenta arrows highlight dual anticlinal plasma membranes (PMs) separated by a cell wall; white arrows indicate the outer periclinal PM. Scale bar, 10 µm. **(b)** Quantified sensor fluorescence intensity ratio between cytoplasm and anticlinal PM domains in root epidermal cells with and without cellulase treatment. Error bars show standard error of the mean (n=30).

(a)

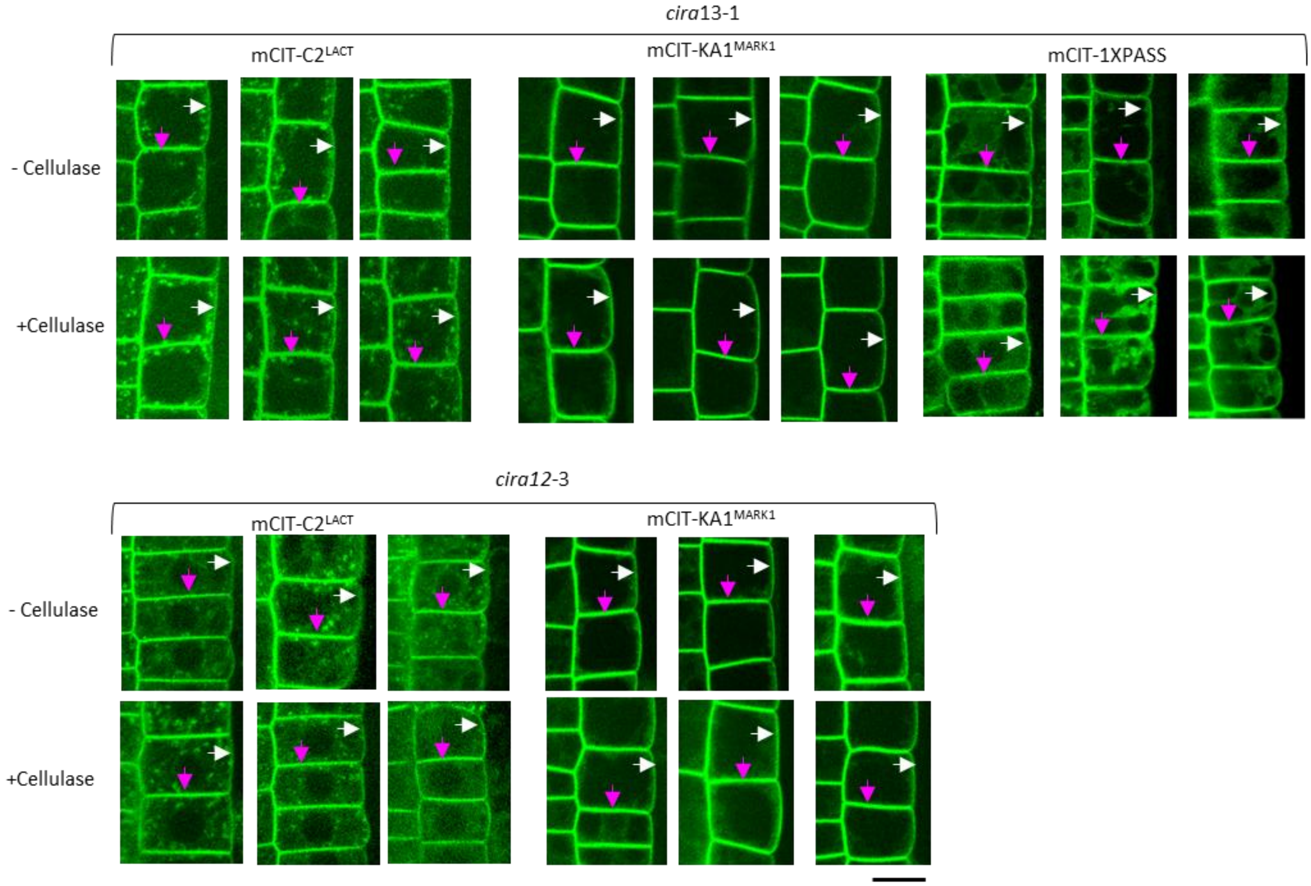
*cira*13-1
mCIT-C2^{LACT}
mCIT-KA1^{MARK1}
mCIT-1XPASS
- Cellulase
+Cellulase
cira12-3
mCIT-C2^{LACT}
mCIT-KA1^{MARK1}
- Cellulase
+Cellulase

(b)

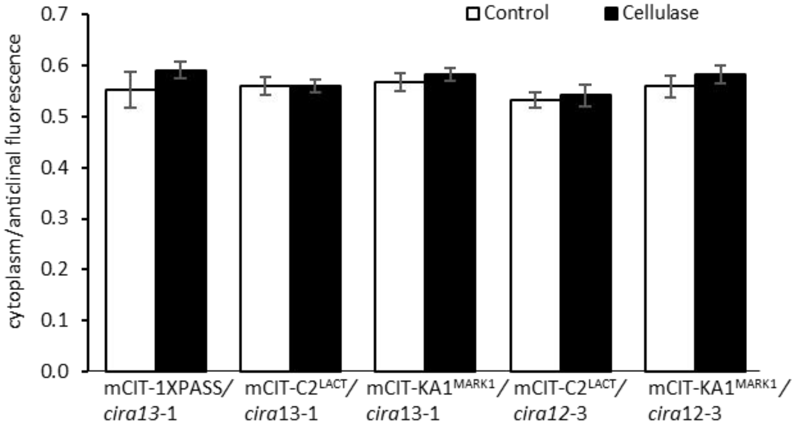
Control
Cellulase
cytoplasm/anticlinal fluorescence
0.7
0.6
0.5
0.4
0.3
0.2
0.1
0.0
mCIT-1XPASS/ *cira13*-1
mCIT-C2^{LACT}/ *cira*13-1
mCIT-KA1^{MARK1}/ *cira*13-1
mCIT-C2^{LACT}/ *cira12*-3
mCIT-KA1^{MARK1}/ *cira*12-3

Fig. S7 External application of lysophospholipids alone does not affect ionic release in wild type seedlings, but lysophosphatidylserine (lysoPS) enhances the basal alamethicin effect. (a) Seedlings were treated with water and various lysophospholipids and tested for ion release. Seedlings treated with water were used as control. **(b)** Seedlings treated with various lysophospholipids and tested for alamethicin-dependent ion release. Seedlings treated with alamethicin were used as control. Values marked by the same letter are not statistically different ($p < 0.05$). **(c)** Alamethicin-dependent ion release was measured as conductivity after incubating WT seedlings with cellulase sampled at various time points (0, 15, 30, 60, and 120 minutes). Following this, the seedlings were washed with water and subsequently treated with different concentrations of lysophosphatidylserine (lysoPS) (0, 20, 50, and 150 µM) and incubated again for 30, 60, and 120 minutes. Error bars show standard error of the mean (n=3). LysoPC, lysophosphatidylcholine; lysoPE, lysophosphatidylethanolamine; lysoPI, lysophosphatidylinositol; lysoPA, lysophosphatidic acid.

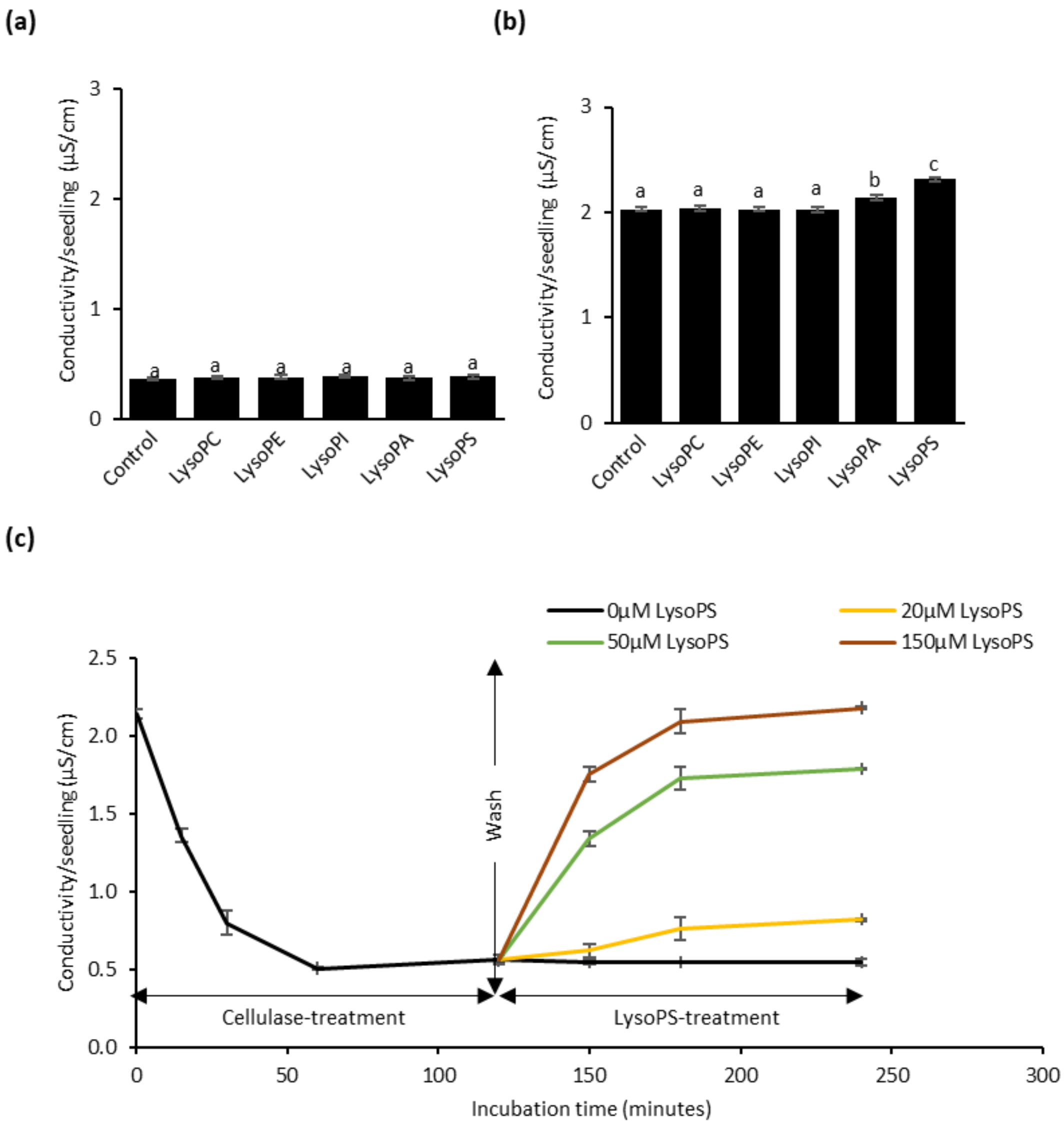
(a)
(b)
(c)
Conductivity/seedling (µS/cm)
Control
LysoPC
LysoPE
LysoPI
LysoPA
LysoPS
a
b
c
0µM LysoPS
20µM LysoPS
50µM LysoPS
150µM LysoPS
Wash
Cellulase-treatment
LysoPS-treatment
Incubation time (minutes)

Fig. S8 External application of anionic phospholipid lysophosphatidylserine (lysoPS) counteracts cellulase-induced resistance to alamethicin (CIRA) in wild type; additional information. **(a**) Root early extension zone epidermis of *Arabidopsis* seedlings expressing distribution probes mCIT-1XPASS, mCIT-C2^{LACT} treated 1 h with 1 % cellulase. Then, the cellulase solution was supplemented with 54 µM lysoPC/lysoPS/lysoPA and gently agitated for 1 h and were analyzed by spinning disc confocal microscopy. LysoPC treated were used as controls. Magenta arrows highlight dual anticlinal PMs separated by a cell wall; white arrows indicate the outer periclinal PM. Scale bar, 10 µm. Images of the seedlings with nucleus stained and those with high cytoplasmic staining than PM were not included. **(b)** Quantified mCIT-1XPASS; and mCIT-C2^{LACT} fluorescence intensity ratio between cytoplasm and anticlinal PM domains in root epidermal cells after cellulase, and lysoPC/lysoPA/lysoPS treatment. Error bars show standard error of the mean (n=30). LysoPC, lysophosphatidylcholine; lysoPA, lysophosphatidic acid; lysoPS, lysophosphatidylserine.

(a)

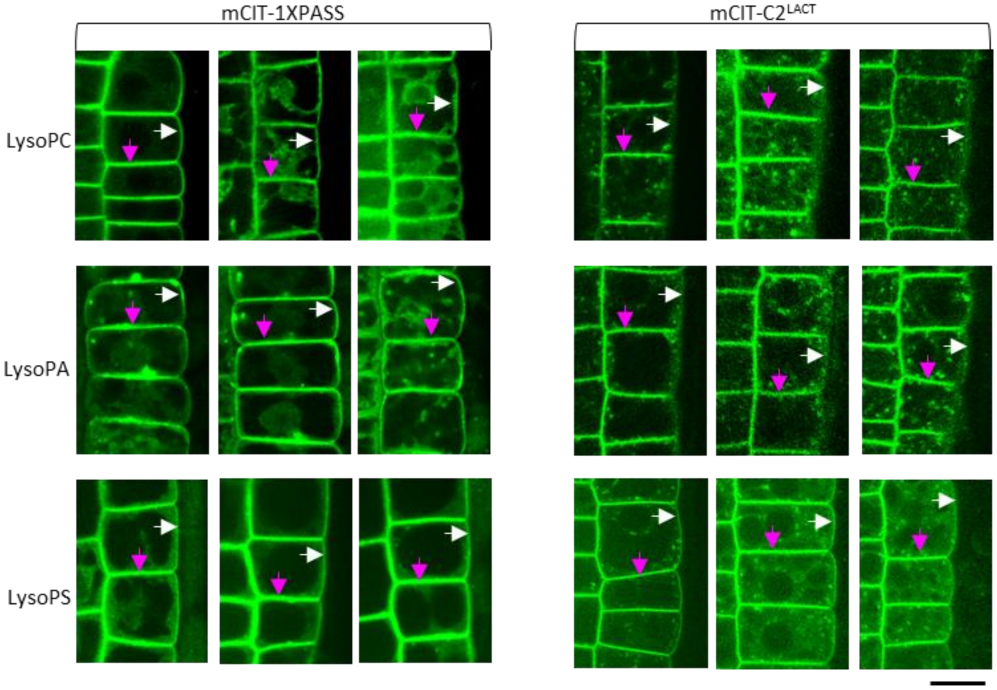

mCIT-1XPASS
mCIT-C2LACT
LysoPC
LysoPA
LysoPS

(b)

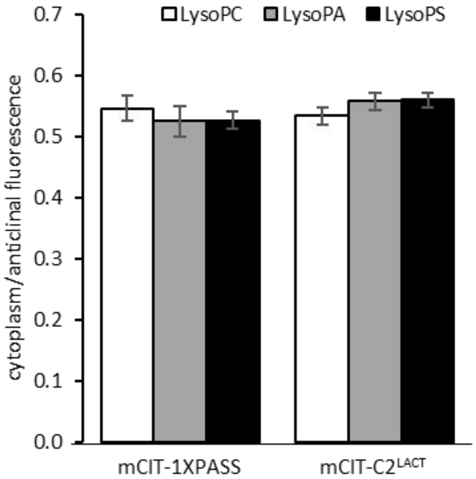

LysoPC
LysoPA
LysoPS
cytoplasm/anticlinal fluorescence
0.7
0.6
0.5
0.4
0.3
0.2
0.1
0.0
mCIT-1XPASS
mCIT-C2LACT

Fig. S9 Clathrin dependent endocytosis and exocyst complex has a minor importance on cellulase-induced resistance to alamethicin (CIRA). Tyrphostin A (T) inhibitor of clathrin dependent endocytosis and endosidin2 (E) inhibitor of exocytosis by binding to an exocyst complex were tested for effects on CIRA in WT and *cira* mutants. Seedlings were pre-treated with T or E for 30 and 120 min, respectively, washed twice in water followed by cellulase for 2 h, washed thrice in water and then tested for alamethicin-dependent ion release. Application of T and E exhibited a minor difference in alamethicin-dependent conductivity in cellulase-treated WT seedlings and had no effect on *cira* mutants. Seedlings pre-treated with and without cellulase were used as controls. Seedlings were grown in ½ MS for 6 days prior to treatments. Values marked by the same letter are not statistically different ($p < 0.05$). Error bars show standard error of the mean (n=3). A, alamethicin; C, cellulase; BFA, brefeldin A.

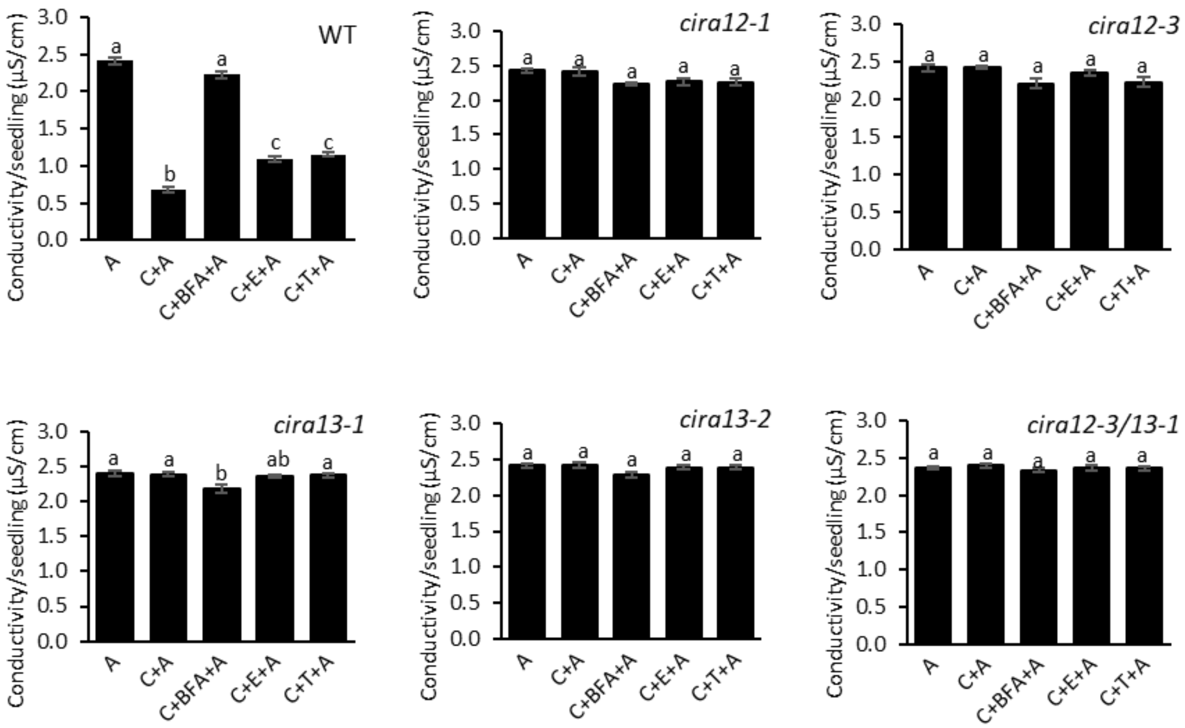

Fig. S10 Cellulase-induced resistance to alamethicin (CIRA) depends on membrane vesicular trafficking; additional information. (a, c) External application of inhibitors of endocytosis, wortmannin (WM), and exocytosis, brefeldin A (BFA) prevent CIRA in wild-type. Root early extension zone epidermis of *Arabidopsis* wild-type (WT) seedlings expressing the distribution probes mCIT-1XPASS, mCIT-C2^{LACT} treated with cellulase and WM **(a)** or BFA **(c)** were analyzed by spinning disc confocal microscopy. Magenta arrows highlight dual anticlinal plasma membranes (PMs) separated by a cell wall; white arrows indicate the outer periclinal PM. Scale bar, 10 µm. **(b, d)** Quantified mCIT-1XPASS; and mCIT-C2^{LACT} fluorescence intensity ratio between cytoplasm and anticlinal PM domains in root epidermal cells after cellulase, and WM/BFA treatment. Error bars show standard error of the mean (n=30).

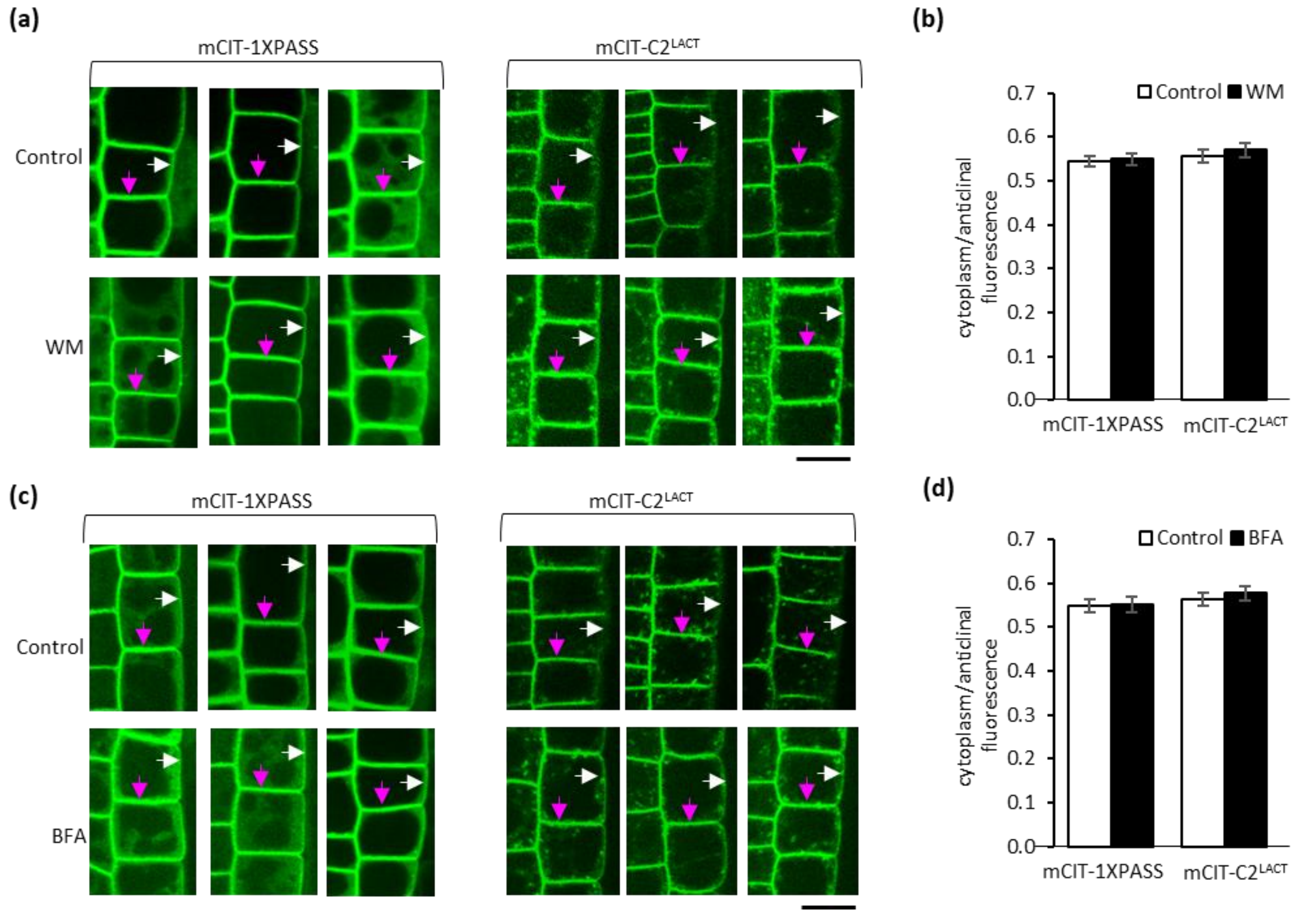

Fig. S11 PHOSPHOLIPASE Dζ inhibitors prevent cellulase-induced resistance to alamethicin (CIRA) in wild-type (WT) but not in *cira12*; additional information. (a) Root early extension zone epidermis of *Arabidopsis* wild-type (WT) and mutant seedlings *cira12-3* expressing the distribution probe mCIT-tagged PS (mCIT-C2^{LACT}) treated with and without cellulase and/or with and without *PLDζ*-inhibitors (VUs) were analyzed by spinning disc confocal microscopy. Magenta arrows highlight dual anticlinal plasma membranes (PMs) separated by a cell wall; white arrows indicate the outer periclinal PM. Scale bar, 10 µm. **(b)** Quantified phosphatidylserine sensor fluorescence intensity ratio between cytoplasm and anticlinal PM domains in root epidermal cells with and without cellulase treatment and/or with and without *PLDζ*-inhibitors. Error bars show standard error of the mean (n=30).

(a)

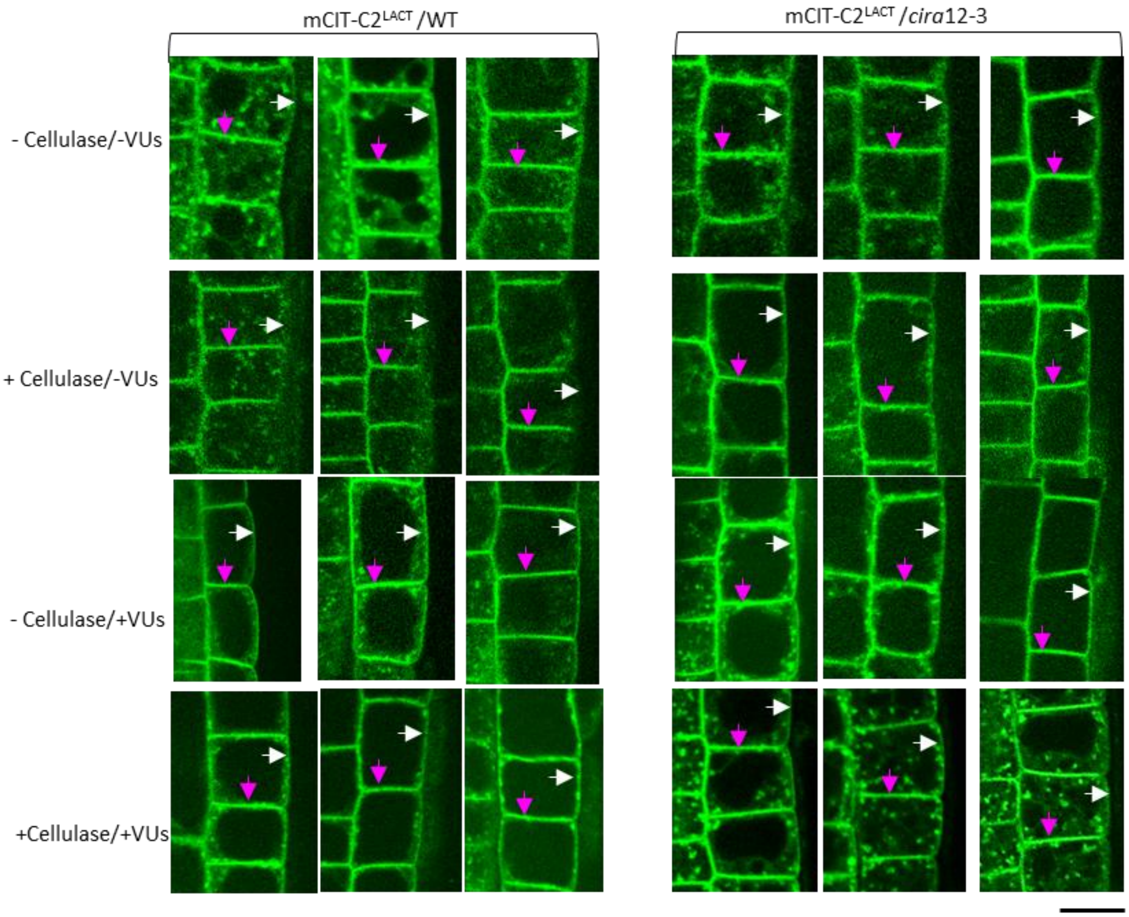
mCIT-C2LACT /WT
mCIT-C2LACT /cira12-3
- Cellulase/-VUs
+ Cellulase/-VUs
- Cellulase/+VUs
+Cellulase/+VUs

(b)

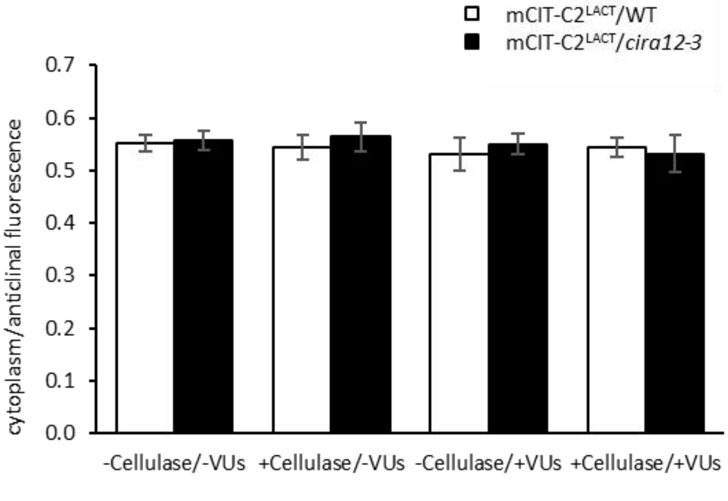
mCIT-C2LACT/WT
mCIT-C2LACT/cira12-3
cytoplasm/anticlinal fluorescence
0.7
0.6
0.5
0.4
0.3
0.2
0.1
0.0
-Cellulase/-VUs
+Cellulase/-VUs
-Cellulase/+VUs
+Cellulase/+VUs

Fig. S12 PHOSPHOLIPASE Dζ inhibitors do not affect *cira13*; additional information. (a) Plasma membrane (PM) of wild-type (WT) seedlings expressing mCIT-1XPASS and *cira13-1* expressing mCIT-C2^{LACT} is not affected by external application of *PLDζ*-inhibitors (VUs). Root early extension zone epidermis of *Arabidopsis* WT seedlings expressing the distribution probe mCIT-tagged phosphatidic acid (mCIT-1XPASS) and *cira13-1* expressing mCIT-tagged phosphatidylserine (mCIT-C2^{LACT}), treated with cellulase and with and without *PLDζ*-inhibitors were analyzed by spinning disc confocal microscopy. Magenta arrows highlight dual anticlinal PMs separated by a cell wall; white arrows indicate the outer periclinal PM. Scale bar, 10 µm. **(b)** Quantified sensor fluorescence intensity ratio between outer periclinal and anticlinal PM domains in root epidermal cells with and without *PLDζ*-inhibitors. **(c)** Quantified sensor fluorescence intensity ratio between cytoplasm and anticlinal PM domains in root epidermal cells with and without *PLDζ*-inhibitors. Ratios have been corrected for the anticlinal signal coming from two membranes in close proximity. Error bars show standard error of the mean (n=30).

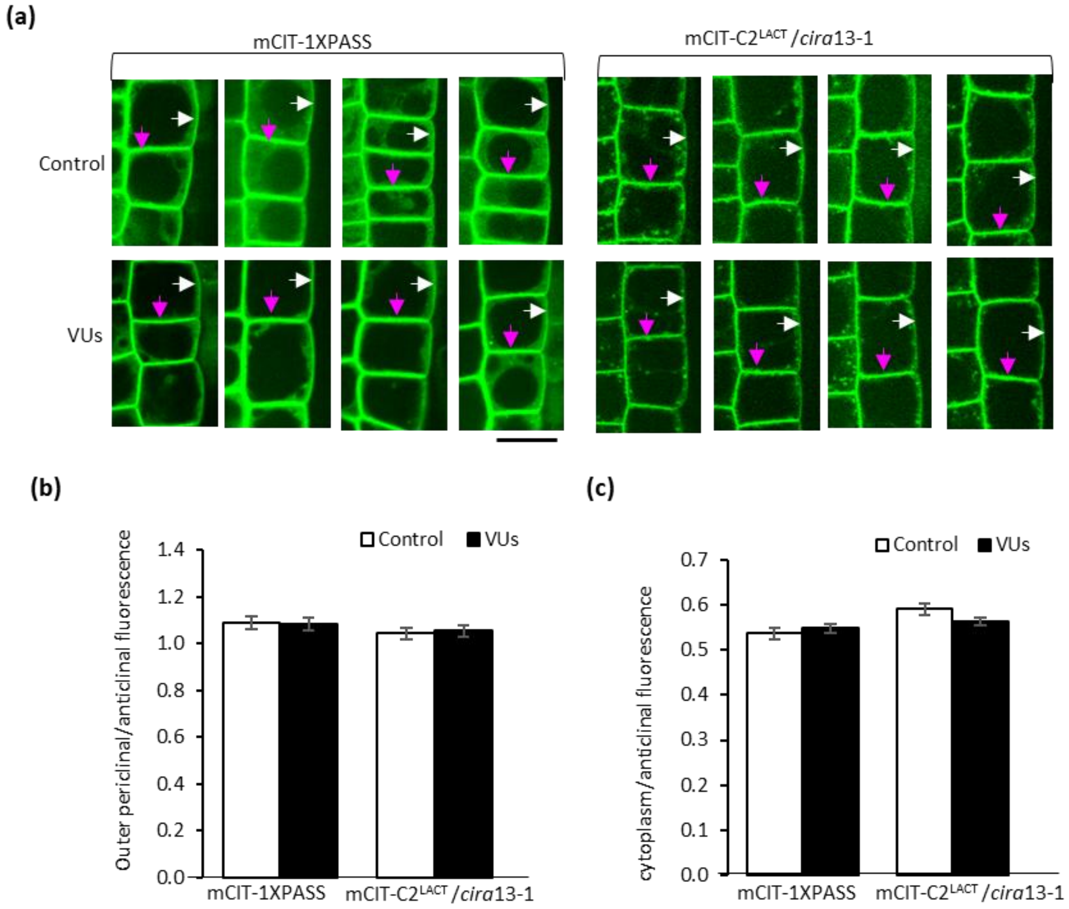

(a)
mCIT-1XPASS
mCIT-C2LACT /cira13-1
Control
VUs
(b)
Control
VUs
Outer periclinal/anticlinal fluorescence
1.4
1.2
1.0
0.8
0.6
0.4
0.2
0.0
mCIT-1XPASS
mCIT-C2LACT /cira13-1
(c)
Control
VUs
cytoplasm/anticlinal fluorescence
0.7
0.6
0.5
0.4
0.3
0.2
0.1
0.0
mCIT-1XPASS
mCIT-C2LACT /cira13-1

Table S1 PCR primers used in this study. LP/RP primers are used for genotyping and F/R primers are used for RT-PCR analysis.

Primer	Sequence
cira12-1 LP	CACGAAAAGCAGAAAGAGGTG
cira12-1 RP	GCTTCTCCTGGAGAAAAATGG
cira12-3 LP	TTGCTCATATTTGCAGGATCC
cira12-3 RP	GCTCAAGCCAGGATTCACTAC
cira13-1 LP	AACCTTAACGCTTTGCTCGG
cira13-1 RP	GGGATGCACGAGAAGACTTG
cira13-2 LP	CCGATTTTAGTCCCTTGCTTC
cira13-2 RP	TTTGACCAAACCTTTGTCCAG
cira13-3 LP	CTTCATGAGCCTTCAGAATGC
cira13-3 RP	CGGCATTTACCTCTGGTACAG
pldz1 LP	TGAAAAGCATGGAAATTTTCG
pldz1 RP	GTGATCGTCTCTGTCTCTCGC
psd2 LP	TTTGCAAAGCGTATCTTGTCC

psd2 RP	CTCCTTTGCCCCTAAATCAAG
pss1 LP	GGGGCAGAACAAAGATGAAAG
pss1 RP	TCATGGTAGGTATCTGGGCAG
LBb 1.3	ATTTTGCCGATTTCGGAAC
LBGABI	ATATTGACCATCATACTCATTGC
12-1 F	TTCAAGGACAAGTGGCTCGC
12-1 R	ATTCAGGTCAGCGGCTTTGA
12-3 F	TTGGGAATGTAGTGGCTGCT
12-3 R	TCAACTCTTCCACGAGCCTC
13-1 F	AACCTTAACGCTTTGCTCGG
13-1 R	GGGATGCACGAGAAGACTTG
13-2 F	CCGATTTTAGTCCCTTGCTTC
13-2 R	TGTTCTCAGCCTCACGGTTC
13-3 F	GTCACACTGCGTTTGAAG
13-3 R	CTCTGCTTCCCAATCTGCCA
